# Two Orders of Magnitude Reduction in Computational Load Achieved by Ultrawideband Responses of an Ion-Gating Reservoir

**DOI:** 10.1021/acsnano.5c06174

**Published:** 2025-10-14

**Authors:** Daiki Nishioka, Hina Kitano, Wataru Namiki, Satofumi Souma, Kazuya Terabe, Takashi Tsuchiya

**Affiliations:** † International Center for Young Scientists (ICYS), National Institute for Materials Science (NIMS), 1-1 Namiki, Tsukuba, Ibaraki 305-0044, Japan; ‡ Research Center for Materials Nanoarchitectonics (MANA), National Institute for Materials Science (NIMS), NIMS, 1-1 Namiki, Tsukuba, Ibaraki 305-0044, Japan; § Department of Applied Physics, Faculty of Advanced Engineering, Tokyo University of Science, Katsushika, Tokyo 125-8585, Japan; ∥ Department of Electrical and Electronic Engineering, Kobe University, Kobe 657-8501, Japan

**Keywords:** Reservoir Computing, Neuromorphic Computing, Ion-Gating Reservoir, Electric-Double Layer Transistor, Iontronics, Information Processing Capacity, Response Speed

## Abstract

The rising energy demands of conventional AI systems underscore the need for efficient computing technologies, such as brain-inspired computing. Physical reservoir computing (PRC), leveraging the nonlinear dynamics of physical systems for information processing, has emerged as a promising approach for neuromorphic computing. However, current PRC systems are constrained by narrow responsive time scales and limited performance. To address these challenges, an ion-gel/graphene electric double layer (EDL) transistor-based ion-gating reservoir (IGR) was developed. This IGR achieves a highly tunable and ultrawide time-scale response through the coexistence of fast EDL dynamics at the ion-gel/graphene interface and slower molecular adsorption dynamics on the graphene surface. Consequently, the system demonstrates an exceptionally broad responsive range, from 1 MHz to 20 Hz, while maintaining a high information processing capacity and adaptability across multiple time scales. The IGR achieved deep learning (DL)-level accuracy in chaotic time series prediction tasks while reducing computational resource requirements to 1/100 of those needed by DL. Principal component analysis reveals the IGR’s superior performance stems from its high-dimensionality, driven by the ultrawideband responses of the EDL along with the ambipolar behavior of graphene. The proposed IGR represents a significant step forward in providing low-power, high-performance computing solutions, particularly for resource-constrained edge environments.

## Graphical abstract

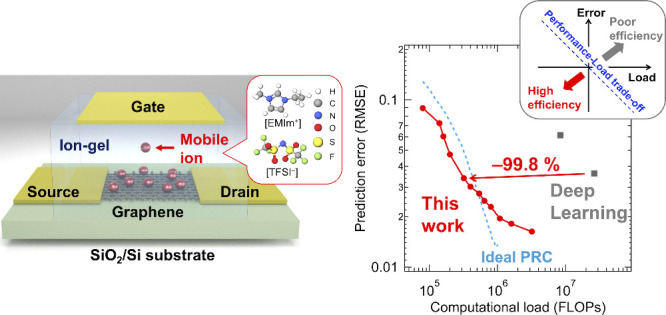

The rapid development of machine learning (ML) technologies, represented by deep learning (DL) and generative artificial intelligence (AI), has significantly increased power consumption, creating a serious social challenge despite the tremendous benefits provided.^1,2^ This high energy demand renders conventional cloud-based computing systems unsustainable and necessitates a shift to low-power alternatives like edge computing, where information is processed locally.^3^ This shift drives the urgent need for high-performance, low-power ML hardware, which current semiconductor technologies cannot meet.^4^ Physical reservoir computing (PRC), a neural network approach leveraging the nonlinear dynamics of materials and devices as computational resources, has attracted significant attention for achieving these goals.^2,5^ Despite exploring various materials and devices for high-performance PRC, no ideal candidate has yet been found.^2,5–36^

Ion-gating reservoirs (IGRs), which operate via ion-gating transistor mechanisms, have demonstrated promising PRC performance due to their diverse drain current responses and high-density electronic carrier tuning.^37–43^ In particular, IGRs based on electric double layer transistor (EDLT) mechanisms exhibit excellent PRC performance driven by nonlinear dynamics in an edge-of-chaos state, although further improvement is needed.^38^ Since EDLT-based IGR has a simple thin film field effect transistor (FET) structure, it has a huge potential for highly integrated PRC devices. However, EDLT-based IGRs face a significant limitation: their temporal state evolution relies on a single, slow relaxation process, resulting in a very narrow responsive range. For example, a one-order decrease in optimal operating speed reduces performance to about one-tenth.^38^ The typical response speed of general EDLTs is slower than 10 Hz (relaxation time τ ≈ 100 ms),^44–48^ restricting EDLT-based IGR applications to low-frequency dynamics (e.g., blood glucose, weather, seismic waves, ship oscillations, etc.). Moreover, even in these low-frequency scenarios, essential high-frequency dynamic features embedded in the time series are lost, making it difficult to perform complex information processing that accounts for the full spectrum of the frequency components present in the data. By introducing high-speed dynamics to EDLTs, it becomes possible to achieve an exceptionally broad operational speed range, far beyond the capabilities of conventional electronic devices. This advancement would not only enable responsiveness across diverse time scales but also allow EDLT-based IGRs to fully utilize their high PRC performance, making them applicable to a wide variety of information events and scenarios. Furthermore, this approach facilitates information processing that comprehensively captures the diverse frequency components within a given time series, thereby not only broadening the range of time series that can be handled but also leading to substantial performance enhancements in specific computational tasks.

Here, we report the development of a wide range of responsive speed EDLT and its demonstration in high-performance PRC applications. Our EDLT comprising monolayer graphene channel and ion-gel electrolyte showed conductance switching with τ of 99 ns at the shortest, leading to the extremely wide response range of four orders. The 6-channel EDLT-IGR achieved extremely high PRC performance in typical benchmark tasks such as nonlinear autoregressive moving average (NARMA) tasks. In predicting the Mackey–Glass equation (a chaotic system), a widely used benchmark task in ML, our EDLT-based IGR achieved the same accuracy as DL while requiring only 1/100 computational of DL. Furthermore, it achieved an extremely high computational efficiency comparable to or even surpassing the theoretical limit of the efficiency estimated from a well-tuned simulated-reservoir computing (RC). Our work paves the way to high-performance, versatile PRC systems with low power consumption and high integration capability.

## Results and Discussion

### Design and Characterization of the Ion-Gel/Graphene-Based EDLT for Reservoir Computing

PRC is an in-material computing framework that treats the spatiotemporal state evolution of physical systems as a virtual neural network for information processing. Utilizing the high-dimensional mapping capabilities of this network structure, inspired by the cerebellum,^49^ PRC is applied to various tasks such as prediction, classification, and anomaly detection (Figure 1a).^5^ To demonstrate PRC using a high-speed EDLT based on an ion-gel/graphene structure, we fabricated a multiterminal EDLT comprising six channels (ch0–ch5) with varying lengths and widths (5–100 μm/30 and 80 μm) and a common gate (Figure 1b). The channels were made from monolayer graphene grown via chemical vapor deposition (CVD) and transferred onto a SiO_2_/Si substrate. Ion-gel (1-ethyl-3-methylimidazolium bis­(trifluoromethanesulfonyl)­imide: EMIm-TFSI) was used as the electrolyte, and a gold foil served as the gate electrode. Upon application of a gate voltage (*V*_G_), mobile ions in the ion-gel formed an electric double layer (EDL) at the graphene interface. This enabled electron or hole doping into the graphene, modulating the channel resistance, and producing an ambipolar drain current (*I*_D_) response, as shown in Figure 1c. According to molecular dynamics (MD) simulations, the diffusion coefficients of the anion (TFSI^–^) and cation (EMIm^+^) in bulk EMIm-TFSI are estimated to be *D* ^–^ = 1.323 × 10^–11^ m^2^/s and *D* ^+^ = 1.865 × 10^–11^ m^2^/s at 300 K, respectively.^50^ Based on these values, the corresponding transport numbers are *t* ^–^ = 0.585 and *t* ^+^ = 0.415. Similar MD calculations^50^ for EMIM-TFSI-based ion gels using a PVDF matrix yield diffusion coefficients of *D* ^–^ = 0.636 × 10^–11^ m^2^/s and *D* ^+^ = 0.771 × 10^–11^ m^2^/s, leading to estimated transport numbers of *t* ^–^ = 0.452 and *t* ^+^ = 0.548, respectively. These results suggest that both anions and cations contribute to the observed electrostatic modulation in our devices, with a slightly higher transport number for the cation, indicating its relatively greater mobility. To mitigate slow relaxation processes typical of graphene-based FETs,^51–53^ pulsed inputs were applied for both *V*_G_ and the drain voltage (*V*_D_), recording *I*_D_ responses for each *V*_G_. The Dirac point (*V*_Dirac_) shifted positively across all channels due to the *p*-type doping induced by *V*_D_, contact potential differences at the gel/graphene and ion-gel/Au interfaces, and charge trapping in the SiO_2_ layer. Variations in charge trapping caused differences in *V*_Dirac_ among channels, leading to distinct nonlinear responses, essential for achieving the high-dimensionality required in RCs.^5,54^ When *V*_G_ < *V*_Dirac_, the graphene exhibited *p*-type behavior, with *I*_D_ decreasing as *V*_G_ increased, while *V*_G_ > *V*_Dirac_ showed *n*-type behavior, with *I*_D_ increasing as *V*_G_ rose. This ambipolar response enhances nonlinearity compared to conventional IGRs, which often rely on unipolar transport mechanisms.^37–43^ Furthermore, the channel length also affects the modulation range of the drain current. Figure S1a shows the normalized transfer characteristics of channels ch0–ch2, where shorter channels exhibit more pronounced current plateaus, leading to a suppressed on/off ratio as seen in Figure S1b. This effect arises from the relative contribution of the gate-independent lead resistance *R*_L_ compared to the gate-dependent channel resistance *R*_C_, and it becomes more prominent in shorter channels where *R*_C_ is smaller. Our model, which accounts for this effect (Figure S2), successfully reproduces both the channel-length-dependent on/off ratio (Figure S1) and the transport characteristics (Figure S3). Such differences in the modulation range across channels contribute to the emergence of distinct nonlinearities for each channel. For detailed device modeling, see Note S1. The *I*_D_–*V*_G_ characteristics of ch1 with DC input (Figure 1d) showed hysteresis and *V*_Dirac_ shifts over multiple cycles. This behavior is attributed to the coexistence of fast relaxation processes from the EDL effect and slower processes such as charge trapping in the SiO_2_ layer (activation energy (*E*_A_) of 22.47 to 390 meV^55–57^ ) and molecular adsorption on the graphene surface.^51–53,58,59^ These characteristics suggest that the device retains information about past inputs, offering both short-term memory (from EDL effects) and long-term memory (from slower processes). Such a combination of multiple memory time scales improves computational performance in PRCs and other MLs, such as long short-term memory (LSTM) networks.^60^ Figure 1e shows the results of Hall measurements performed on an EDLT with an ion-gel and Au foil applied to a Hall bar-type graphene channel (Figure S4). The ambipolar carrier injection process characteristic of graphene’s Dirac cone was clearly observed outside the Dirac point region, although near *V*_Dirac_, *p*-type, and *n*-type regions coexisted, complicating measurements of carrier density and mobility. As *V*_G_ deviated from *V*_Dirac_, a gradual decrease in mobility was confirmed, caused by carrier scattering under high electric fields, contributing to the plateau observed in the transfer curves in Figure 1c and d.^61^

**Figure 1:**
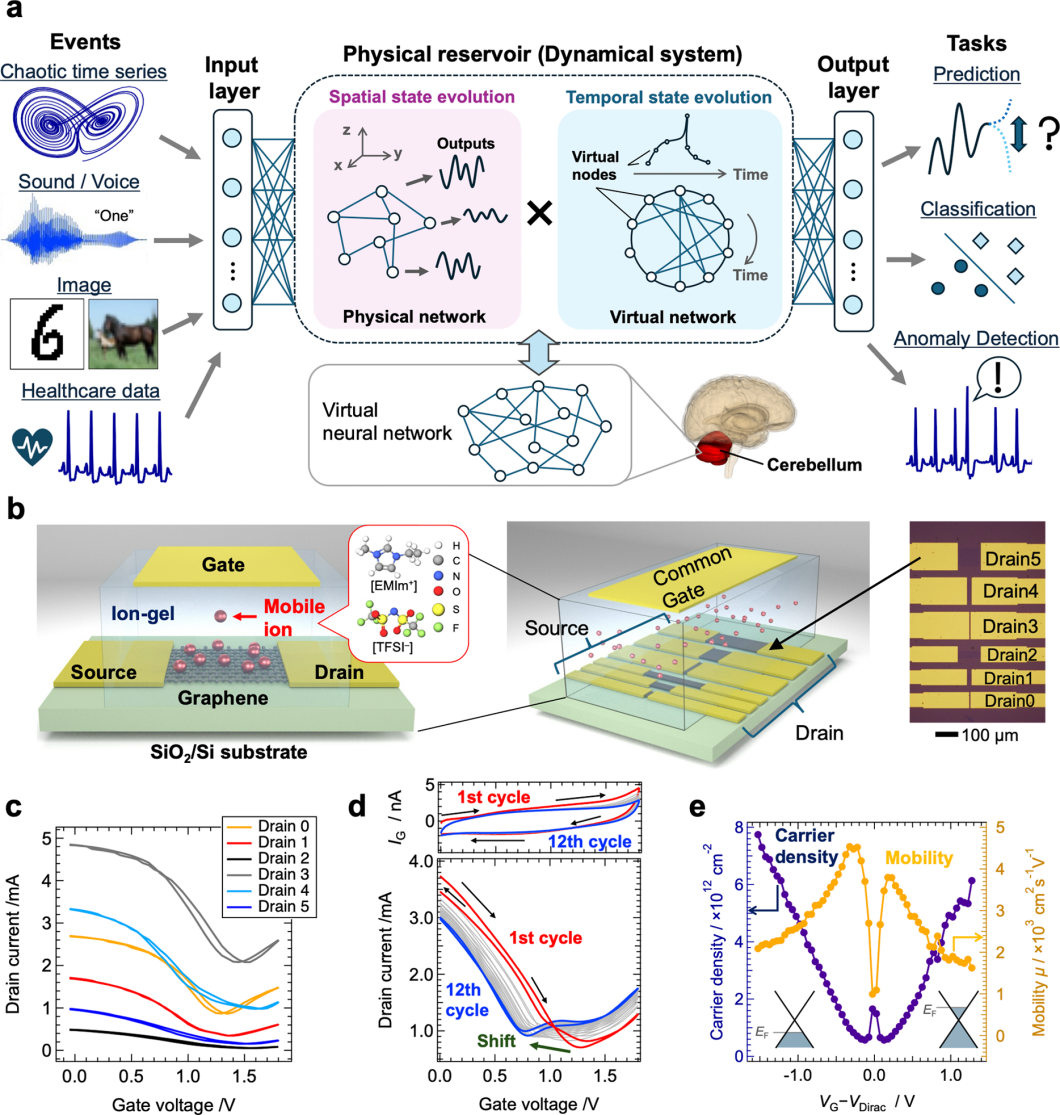
Schematic of PRC and the ion-gating reservoir developed in this study. **a**, A schematic diagram of PRC, which interprets the spatiotemporal state evolution of a physical system as a virtual neural network to perform various information processing tasks. The images and time-series data shown in the figure are based on data sets commonly used in the field of ML.^62–65^ Additionally, the schematic diagram of the brain was created using “BodyParts3D”.^66^ **b**, Cross-sectional and overall schematic diagrams of the EDLT-based IGR composed of an ion-gel and monolayer graphene. The inset shows an optical microscope image of the graphene channel with the source and drain electrodes. **c**, Transport characteristics of the device measured with pulsed *V*_G_ input and **d**, DC *V*_G_ input. **e**, Carrier density and mobility as functions of *V*_G_ obtained from Hall measurements conducted on a Hall bar-type graphene channel.

### Ultra-Fast and Wideband Relaxation Times in Ion-Gel/Graphene EDLTs

In this study, we utilized ion-gel, a commonly used electrolyte. However, by optimizing the operating conditions, as described later, we achieved significantly faster operation than the previously reported time constants.^42,44–48^ Figure 2a and b presents an enlarged and overall view, respectively, of the *I*_D_ response to a pulsed *V*_G_ input for ch1. When *V*_G_ was switched from a base voltage (*V*_b_) of 1.1 V to an input voltage (*V*_in_) of 0 V, the drain current increased sharply from 0.5 to 1.55 mA due to *p*-type doping. The τ, defined as the time it takes for *I*_D_ to change by 1 – 1/*e* (63.2%),^42,48^ was measured at 260 ns. Figure 2c shows the *I*_D_–*V*_G_ curve measured with a pulsed *V*_G_ input. The variation in *I*_D_, when *V*_G_ is switched from 1.1 to 0 V (indicated by the red line), closely aligns with the current variation range observed in the *I*_D_ response to a single *V*_G_ pulse, shown in Figure 2b. This confirms that the high-speed switching behavior of the EDLT aligns with the device’s transfer curve. Figure 2d shows the dependence of τ on *V*_in_ and *V*_b_. As both *V*_in_ and *V*_b_ decrease, τ tends to decrease, with the fastest τ (99 ns) observed under the conditions shown in the right inset. To support the interpretation that the observed ∼100 ns response originates from electric double layer dynamics, we performed numerical simulations based on the Nernst–Planck–Poisson (NPP) equations, incorporating electrostatic screening by mobile ions.^67^ The simulations used physical parameters consistent with the actual device, including the ion gel thickness (500 μm), ionic conductivity (∼6 mS/cm). Importantly, these simulations explicitly capture Debye-scale electrostatic relaxation near the graphene interface rather than bulk-limited ionic transport, which typically governs slower EDL formation. As a result, a comparable switching time was quantitatively reproduced (Figure S5), confirming that the fast response can be explained by ion migration and electrostatic relaxation at the Debye scale (see Note S2 for details). This result supports the notion that the switching behavior is governed by localized interfacial dynamics, enabling significantly faster operation than would be expected from conventional bulk-based models.

**Figure 2:**
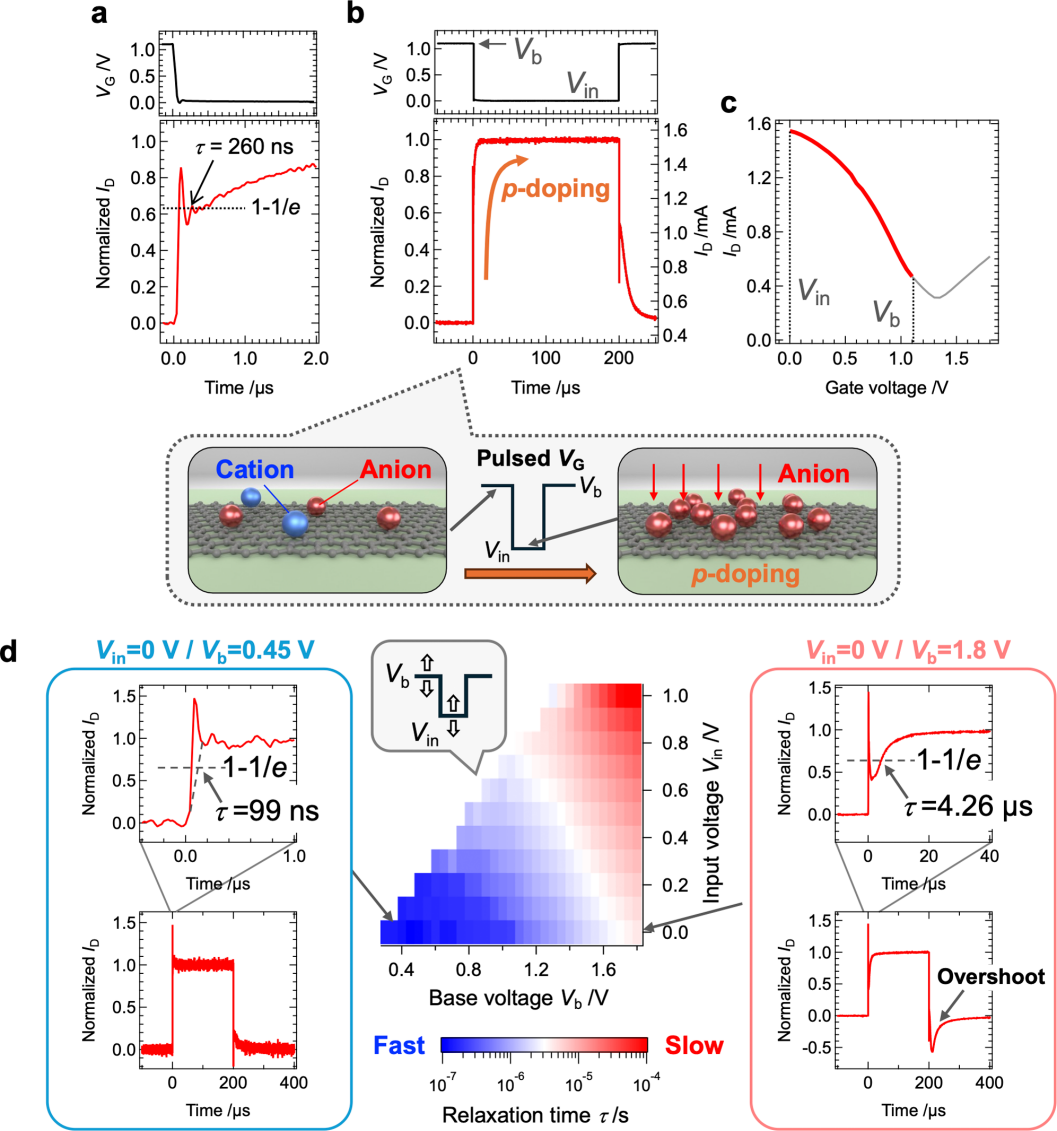
Ultrawideband responsive characteristics of ion-gel/graphene EDLT. **a**, Enlarged view of the *I*_D_ response to a pulsed *V*_G_ input with *V*_b_ = 1.1 V and *V*_in_ = 0, and **b**, overall view. The inset illustrates a schematic of *p*-type doping in the graphene channel induced by the EDL effect, corresponding to the changes in *I*_D_ in response to the *V*_G_ input. **c**, Transport characteristics of the device measured with pulsed *V*_G_ input. The red-highlighted region shows *I*_D_ within the *V*_G_ range of 0 to 1.1 V. **d**, Dependence of relaxation time on *V*_in_ and *V*_b_. The inset shows *I*_D_ responses under specific conditions.

These results suggest that the IGR utilizing the ion-gel/graphene-based EDLT achieves significantly faster operation as a PRC device compared with conventional IGRs. Despite the EDL capacitance calculated from Hall measurements showing little dependence on *V*_G_ (Figure S6 and Note S3), the significant changes in τ, spanning 4 orders of magnitude, indicate the involvement of slower relaxation processes alongside fast EDL relaxation. The dramatic increase in relaxation time with rising *V*_in_ and *V*_b_ is considered to primarily reflect the dominant influence of slower relaxation mechanisms, such as the adsorption/desorption of EMIm^+^ ions on the graphene surface with *E*_A_ of 110 to 160 meV,^58,59,68^ compared to the aforementioned fast EDL charging/discharging processes. The coexistence of slow and fast relaxation dynamics in this IGR, along with their tunability via input *V*_G_ conditions, suggests the potential to overcome the critical limitation of the narrow responsive range typically associated with conventional PRC systems. Furthermore, the device response not only is characterized by a wide range of time scales but also exhibits unique behaviors favorable for reservoir computing. Notably, under conditions spanning *V*_Dirac_, overshoot behavior corresponding to the V-shaped transfer curve of graphene was observed, as shown in the left inset. This behavior resembles inhibitory postsynaptic potentials, representing complex pseudosynaptic responses that are valuable for information processing in PRCs.^52^ Table 1 presents a benchmark comparison of key parameters for various ion-gated devices based on ion gels.^69–73^ Compared to devices with organic semiconductor channels,^69,72,73^ our device—which employs graphene, a semimetal, as the channel material—exhibits a lower on/off ratio but significantly faster maximum operating speed. The pronounced nonlinearity arising from the characteristic V-shaped transfer curves, along with the wide dynamic range of operating time scales described above, indicates that this material system is well suited for PRC. In addition, the relatively high carrier mobility observed in our graphene channel suggests that damage to the graphene during fabrication is likely to be limited.

**Table 1:** Benchmark Comparison of Key Parameters for Ion-Gating Devices Based on Ion Gels^69–73^

electrolyte	channel	capacitance (μF/cm^2^)	mobility (cm^2^V^–1^s^–1^)	on/off ratio	turn-on voltage (V)	maximum operating frequency	ref
[EMIM][TFSI]-based gel	P3HT	43	1.1	∼10^5^	–0.7	∼1 kHz	^69^
[BMIM][PF_6_]-based gel		41	1.0	∼10^5^	–0.8	∼100 Hz	
[EMIM][OctOSO_3_]-ased gel		38	1.2	∼10^4^	–1.2	∼1 Hz	
[EMIM][TFSI]/PS–PMMA-PS-based gel	Monolayer graphene	5.17	Hole: 26	-	-	-	^70^
			Electron: 20				
	Trilayer graphene		Hole: 1131	-	-	-	
			Electron: 362				
[EMIM][TFSI]-based gel	Monolayer graphene	7.29	Hole: 852	11.5	-	-	^71^
			Electron: 452				
[EMIM][TFSI]-based gel	P3HT	∼20	1.8	∼10^5^	–2.7	10 kHz	^72^
[EMIM][TFSI]/SMS-based gel	PQT-12	30.1	1.77	∼5 × 10^4^	–0.2	2.4 kHz	^73^
[EMIM][PF6]/SMS-based gel		23.7	0.3	∼10^5^	–0.7	210 Hz	
[EMIM][TFSI]-based gel	Monolayer graphene	∼1	Hole: 2090–4535	9.91	-	∼10 MHz	This work
			Electron: 1624–3798				

The ultrawide temporal response observed in the IGR arises from the interplay between fast and slow ionic dynamics at the ion-gel/graphene interface. At low gate voltages, the response is dominated by rapid EDL formation occurring within submicrosecond time scales, as supported by experimental observations and numerical simulations (see Note S2). As the applied voltage increases (in both *V*_in_ and *V*_b_), slower interfacial processes gradually become more involved, leading to a progressive broadening of the temporal response. This slower component is likely associated with field-induced molecular interactions at the interface, such as the adsorption of EMIM-based cations.^58,59,68^ The resulting combination of fast and slow responses enables continuous modulation of device dynamics over multiple time scales, providing a flexible platform for temporal information processing in physical reservoir computing. Such multitime scale characteristics, which are advantageous for PRC, can also be extended to ion-gating devices with high integrability, such as vertical organic electrochemical transistors (vOECTs).^74–78^ In general, the operation speed of vOECTs is governed by the mobility of ions within the mixed ionic-electronic conducting channel, and time constants as fast as τ = 45 μs have been reported.^74^ Therefore, as shown in Figure S7a, stacking channel materials with different ion diffusion coefficients enables the design of devices with arbitrary time scale responses. Furthermore, the use of ambipolar or anti-ambipolar channel materials can achieve enhanced nonlinearity.^75,76^ Since the operation speed of vOECTs depends on the channel area,^77,78^ the use of layered graphene channels allows for the integration of multiple devices with distinct time constants by varying the channel area across the stacked layers, as shown in Figure S7b. In this case, the operation speed is determined by both the channel area and the mobility of the mobile ions within the graphene layers. While the present study employs monolayer graphene, where ion trapping within the layer is not observed (only surface adsorption is involved), such trapping effects in multilayer configurations may offer additional control over the temporal response. These insights and device concepts are well aligned with the current vision for iontronics such as vertical ion-gating transistor (IGVT) technologies, which are increasingly recognized as promising candidates for energy-efficient and scalable neuromorphic systems.^74–79^ Recent perspectives have emphasized critical directions such as the development of patternable solid electrolytes, improved models of mixed ionic–electronic transport, and the integration of IGVTs into multimodal synaptic architectures.^79^ Our demonstration of tunable multitime scale dynamics and efficient nonlinear mapping using ion-gel/graphene-based EDLTs represents a material- and device-level realization of these forward-looking concepts. This is particularly important in multimodal information processing, where not only the ability to handle signals across diverse time scales but also the effective extraction of features spanning a wide frequency spectrum is essential. These results suggest that extending the current IGR approach to IGVT architectures could offer a promising pathway toward highly integrated, low-power neuromorphic systems capable of multimodal processing and dynamic adaptation.

To further evaluate the impact of channel length on device response speed, we fabricated eight ion-gating transistors with graphene channels of varying lengths (from 5 to 1000 μm) and a fixed channel width of 30 μm, as shown in Figure S8 (a) to (h). The relaxation times were extracted from their pulse response characteristics. Figure S8 (i) presents the channel resistance as a function of channel length, revealing a linear increase in resistance with increasing length. Figure S8 (j, k) shows the normalized drain voltage responses to pulsed gate inputs for each device, where longer channels exhibit progressively slower responses. This trend is clearly illustrated in Figure S8 (l), which confirms a systematic increase in the relaxation time with increasing channel length. These variations in response speed are primarily attributed to differences in the channel resistance of graphene; higher resistance leads to a slower charging and discharging process of the EDL, driven by the gate voltage. These findings demonstrate that the geometric structure of the graphene channel—particularly its length—is a critical design parameter in tuning the EDL formation rate and the temporal response characteristics of the device. Accordingly, the present IGR design, composed of channels with diverse geometries (Figure 1b), is especially suited for multitime scale operation, suggesting its strong potential to effectively extract features across a wide range of frequency components embedded in the input time series.

### Information Processing Scheme in Ion-Gel/Graphene-based IGR

To evaluate the information processing capability of the developed IGR, benchmark evaluations were conducted within the framework of PRC, which uses the spatiotemporal dynamics of a physical system as a virtual neural network (Figure 1a). The reservoir (the physical system) performs high-dimensional feature mapping similar to neural networks, with the system’s nonlinear dynamics reducing computational costs compared to simulation-based MLs.^5,80^ The developed IGR, which consists of multiple channels with various geometries (Figure 1b), exhibits diverse and distinct responses to a common gate input, enabling the mapping of input information into a highly dimensional feature space. Figure 3(a) shows input gate pulses with varying amplitudes *V*_in_, while Figure 3(b) to (h) displays the corresponding current responses from each terminal. Due to differences in characteristics such as time constants, resistance values, and Dirac point voltages among the channels, each responds differently even under identical gate input conditions—showing variations in rise time, relaxation behavior, and current level. As a result, the device as a whole realizes rich spatiotemporal responses, allowing it to function as a physical reservoir that performs nonlinear, highly dimensional transformations of input signals. Such spatiotemporal diversity in internal states (called reservoir states) can be leveraged in a downstream readout layer by using linear regression or similar techniques to learn and extract relevant information, enabling applications in standard tasks such as time-series prediction and classification.

**Figure 3:**
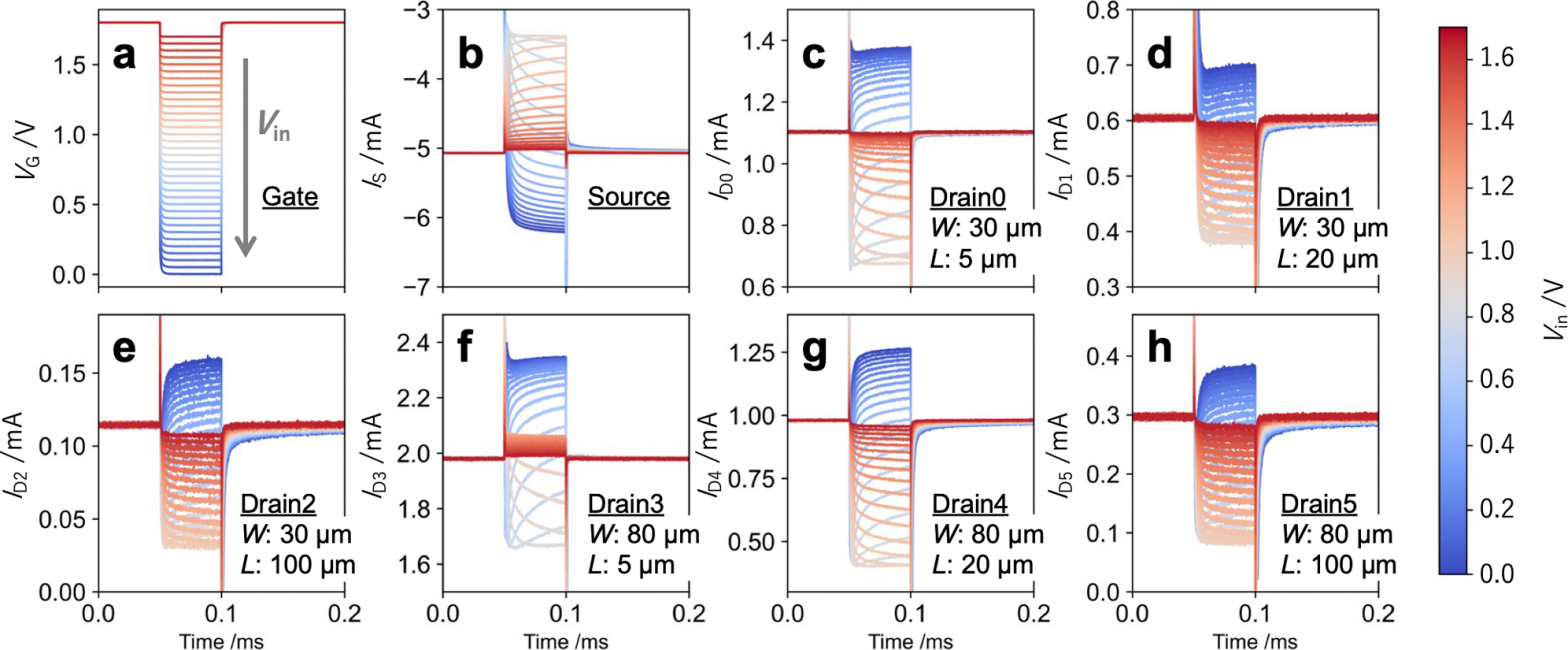
Multichannel responses to gate pulse inputs. **a**, Pulse input signals of varying amplitude *V*_i__n_ applied to the common gate terminal. **b**, corresponding source current response and **c**–**h**, drain current responses to these pulse inputs.

Figure 4a illustrates the IGR’s information processing scheme. Input data *u*(*k*) are converted into pulse voltage signals applied to the gate terminal, where *k* represents discrete time. The pulse *V*_G_ signal had a base voltage of 1.8 V, with intensities ranging from 0 to 1.8 V and a 50% duty cycle. The pulse period (*T*), a key PRC hyperparameter influencing the system’s temporal information processing,^16,29,80–82^ was varied from 1 μs to 50 ms. To enhance the IGR’s memory capacity, delayed inputs *u*(*k* – *d*_in_) to *u*(*k* – 5*d*_in_) were converted into step-like drain voltages *V*_D1_ to *V*_D5_, respectively, and applied to the device (Figure 4a), with *d*_in_ set to 1 or 2 for the tasks. These drain voltages ranged from 0 to 1 V, while a constant (*V*_D0_ = 0.5 V) was applied to drain 0. Figure 4b shows the random input *V*_G_ (top), and the corresponding *I*_D0_ (middle) and *I*_D1_ (bottom) responses. The *I*_D0_ response, under the constant *V*_D0_, reflects mainly *V*_G_, while the *I*_D1_ response, incorporating delayed inputs, displays more complex dynamics, but *I*_D0_ behavior highlights the interplay between rapid carrier injection (due to EDL charging/discharging) and slower relaxation processes (e.g., molecular adsorption and charge trapping).^51–53,58,59^ Fluctuations in *I*_D0_ during *V*_G_ pulse intervals (blue arrows) indicate the system’s sensitivity to both current and past inputs, enhancing its ability to express complex dynamics across diverse conditions. In addition to the six *I*_D_ responses, the gate current (*I*_G_) and source current (*I*_S_) were used as physical nodes, further increasing the system’s dimensionality.^6,43^ To fully exploit the dynamic behavior of these currents, time multiplexing was applied to generate 10 virtual nodes from each current response at discrete time points (Figure 4c). This approach produced 80 reservoir states from a single IGR. By incorporating inverted input signals (*u*_inv_(*k*) = *u*_max_ – *u*(*k*)), an additional 80 reservoir states were generated, resulting in a total of 160 states.^39^ The reservoir output *y*(*k*) was calculated as a linear sum of reservoir states *X*_*i*_(*k*) and readout weights *w*_*i*_, as shown below:

1 $$y\left(k\right)=\overset{N}{\underset{i=1}{\sum }}w_{i}X_{i}\left(k\right)+b$$

where *N* (=160) is the reservoir size, and *b* is the bias. The readout weights were trained via ridge regression to minimize the error between *y*(*k*) and target *y*_t_(*k*).

**Figure 4:**
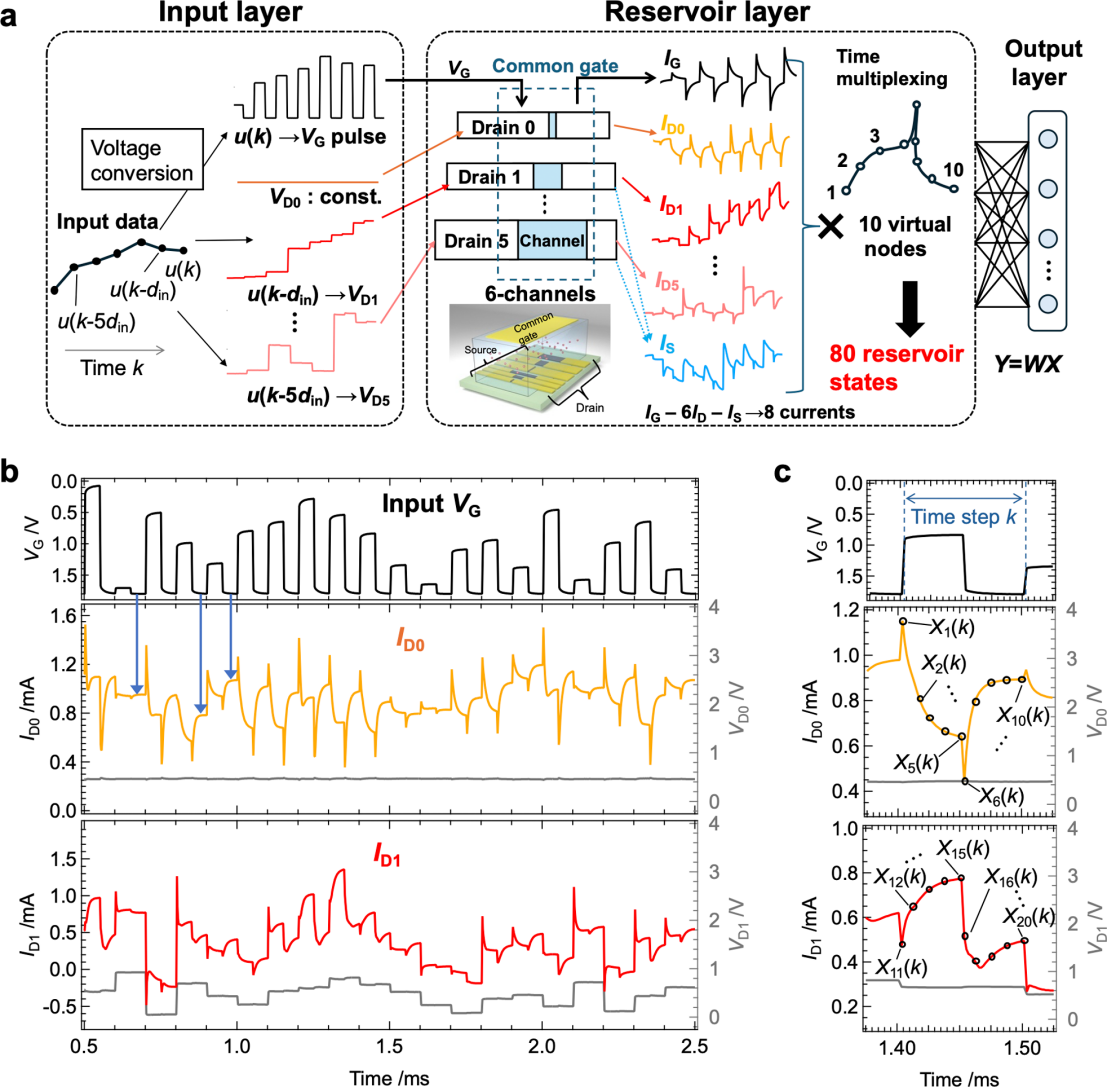
Information processing in the IGR. **a**, Schematic of the PRC scheme utilizing the IGR. **b**, Example of *I*_D0_ (middle panel) and *I*_D1_ (bottom panel) responses to the input *V*_G_ (top panel). Blue arrows indicate changes in *I*_D0_ due to slow dynamics during the *V*_G_ pulse intervals. **c**, Example of the method for acquiring virtual nodes. As shown, 10 current values were obtained as virtual nodes from the real-time domain, corresponding to a single discrete time step.

### Performance Evaluation of the IGR Using the NARMA2 Task

First, we performed the NARMA2 task, a widely used PRC benchmark,^14–20,28,35,38,41,43^ and required the reservoir to reproduce and predict the second-order nonlinear dynamical system described by eq 2, necessitating both nonlinearity and memory:

2 $$y\left(k+1\right)=0.4y\left(k\right)+0.4y\left(k\right)y\left(k-1\right)+0.6u^{3}\left(k\right)+0.1$$

Here, *u*(*k*) is a random sequence ranging from 0 to 0.5. As eq 2 lacks long-term delay terms, PRCs with modest memory can perform this task reasonably well. However, high accuracy requires strong nonlinearity, as seen in chaotic states in spin-wave interference RCs^15,35^ and edge-of-chaos states in diamond-based IGRs.^38^ Using the scheme in Figure 4a, we set *d*_in_ = 1, applying *u*(*k*) to the gate and *u*(*k* – 1) to *u*(*k* – 5) to drains 1 to 5. A 2500-step random sequence was input, with 100 steps for reservoir washout, 1600 steps for training, and 800 steps for testing.

To further analyze the computational performance, we evaluated the Information Processing Capacity (IPC), an extension of Memory Capacity (MC), that characterizes nonlinear capacity and overall computational ability.^83,84^ Total IPC (*C*_tot_) is the sum of order-specific partial capacities (*C*_*n*_), where *n* represents the degree of nonlinearity:

3 $$C_{\mathrm{tot}}=\underset{n=1}{\sum }C_{n}$$

Linear capacity (*C*_1_) is assessed by reconstructing delayed inputs *u*(*k* – *d*) from reservoir states, while nonlinear capacity is based on generating *y*_*t*_(*k*) = ∏*P*_*n* ^’^_ (*k*) using an *n’*-th order polynomial *P*_*n’*_ (Gram-Schmidt polynomials in this study),^84^ where *n* = ∑*n*’. IPC, a task-independent metric, indicates higher computational power with a larger *C*_tot_ including higher-order terms.^83,84^ By evaluating the IPC of a reservoir, one can quantitatively assess its ability to reference past input information and perform nonlinear transformations of various orders. Thus, analyzing the IPC under different input conditions and device structures provides practical guidelines for optimizing the operating conditions and design of reservoirs tailored to specific tasks or capable of handling a wide range of computational demands. In particular, comparing the task performance with the order-specific components of IPC under varying conditions enables a quantitative understanding of the memory and nonlinearity required for a given information processing function. For details on the calculation procedure of IPC, see Methods section, Note S4, Figures S9–S11 and Tables S2–S3. Figure 5a shows total IPC (color-coded by *n*) and the normalized mean squared error (NMSE) for the NARMA2 task as a function of *T*. As shown in Figure S12, the NMSE was calculated over all time steps of the test data set. For details on the calculation of NMSE, see the Methods section and Note S5. IPC is initially low but increases with *T*, peaking at (*T* = 50 μs), driven by higher-order terms (*n* ≥ 4). NMSE trends align with this behavior (inset, Figure 5a). Figures 5b and 4c reveal no significant correlation between the linear capacity *C*_1_ and NMSE, but a strong correlation between the nonlinear capacity (*C*_tot_–*C*_1_) and NMSE. This suggests that IGR’s superior nonlinearity drives its high NARMA2 performance. Additionally, *C*_1_, ranging from 7 to 9, suffices for this task, explaining the lack of correlation between *C*_1_ and NMSE across all *T*. The reservoir output and target under the optimal condition (*T* = 100 μs) are shown in Figure 5d, with NMSEs of 5.73 × 10^–3^ during training and 7.35 × 10^–3^ during testing, indicating low errors. Figure 5e positions our system among the top-performing PRCs, ranking second for the NARMA2 task. Figure 5f compares device operation speed and NMSE across PRCs, showing that while the IGR slightly trails the opt-magnonic-RC (which is limited to GHz-scale events and lacks versatility) it maintains high performance over a much broader operational speed range. Notably, the IGR achieves NMSE < 0.03 over a *T* spanning 4 orders of magnitude (1 μs to 50 ms), corresponding to a high prediction accuracy (*r* ^2^ > 97%, *r* ^2^ = 1-NMSE). Generally, PRCs with a single characteristic time scale often lose response diversity when mismatched with input time scales, reducing the computational ability.^16,29,81,82^ In contrast, the IGR integrates multiple relaxation processes with varying time scales (fast and slow dynamics), achieving high *C*_tot_ and computational performance across a wide speed range. This versatility is ideal for edge AI devices requiring time-series processing at different scales, underscoring the potential of IGR-based edge AI systems for efficient on-site information processing.

**Figure 5:**
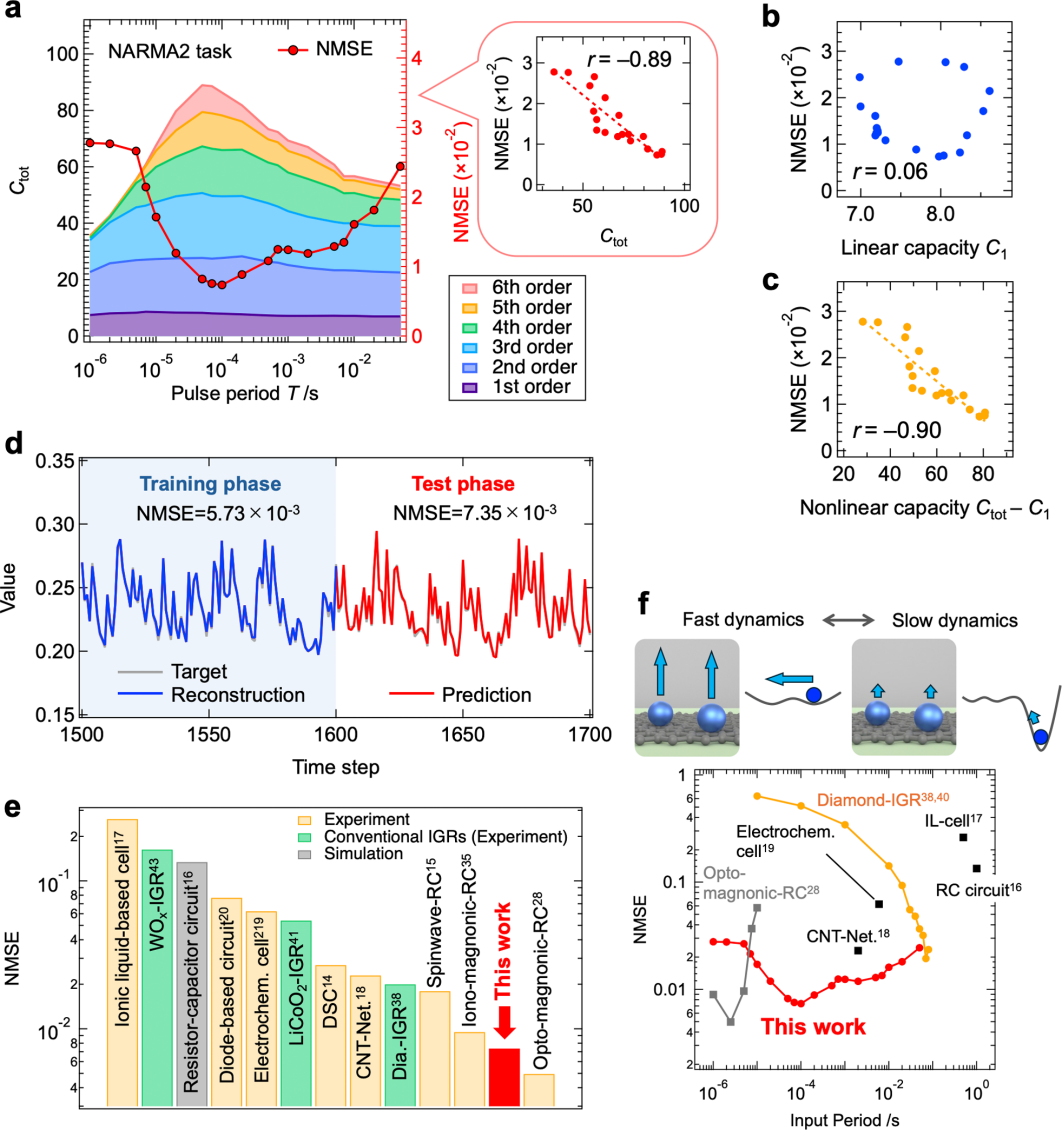
NARMA2 task performed by the IGR. **a**, Dependence of IPC (left axis) and NMSE during the test phase (right axis) on the pulse period. The inset shows a scatter plot of total capacity and NMSE, with *r* and the red dashed line representing the correlation coefficient and linear fitting curve, respectively. Scatter plot of NMSE versus **b**, linear capacity, and **c**, nonlinear capacity. **d**, Prediction output (red line) and target (gray line) during the training and test phases for the IGR operated under optimal conditions (*T* = 100 μs). **e**, Comparison of NMSE during the test phase with other physical reservoirs.^14–20,28,35,38,41,43^ DSC and CNT refer to dye-sensitized solar cells and carbon nanotubes, respectively.^14,18^ **f**, Relationship between device operating speed and NMSE. The operating time of ‘Opt-magnonic-RC’ is displayed as the product of the input pulse period and the number of accumulation cycles. ‘RC circuit’ and ‘IL-cell’ represent a resistor-capacitor circuit and an ionic liquid-based cell, respectively. The plot in the range of 10^–6^ to 10^–3^ s for the diamond-IGR results corresponds to evaluations performed using devices equivalent to those reported in ref 40.

Furthermore, to investigate the influence of graphene’s ambipolar behavior on information processing performance and reservoir dimensionality, we evaluated the input-voltage dependence of IPC and effective dimensionality using newly fabricated devices. In this experiment, the gate pulse period *T* and base voltage *V*_b_ were fixed at 100 μs and 1.8 V, respectively, while the minimum pulse voltage *V*_in_ was varied from 0.0 to 1.6 V using random waveform inputs. As a result, the dynamic range of the input gate voltage, defined as Δ*V* = |*V*_b_ – *V*_in_|, was tuned from 0.2 to 1.8 V. This effectively allowed us to select the operating region of the device’s transport characteristics used for computation, as illustrated in Figure S13, thereby enabling a practical evaluation of how ambipolar behavior contributes to computational performance. In IGRs, the transport characteristics under static conditions correspond to the mapping function of the underlying dynamic system and, thus, have a significant impact on performance. In dynamic operation, reservoir state evolution follows ***x*** (*k*) = ***f*** [*u*(*k*), ***x*** (*k* – 1)], where the system state at time *k* is determined by a function ***f*** of the current input *u*(*k*) and the past state *x*(*k* – 1). Figure S14a shows a 3D plot of *X*_5_(*k*) versus *u*(*k*) and *X*_5_(*k* – 1) under the condition Δ*V* = 1.8 V (where *X*_5_ is a reservoir state derived from drain 0; its spatial location is indicated in Figure 4c). The resulting curved surface can be interpreted as a partial representation of the mapping function in the dynamic regime, exhibiting strong nonlinearity that reflects the ambipolar behavior of graphene. In contrast, another state *X*_6_, which exhibits spike-like behavior, shows a qualitatively different response under the same condition, as illustrated in Figure S14b. These diverse mapping functions arising from ambipolar transport enhance the expressive power of the system, enabling it to produce a wide range of nonlinear responses depending on both current inputs and past states. On the other hand, when Δ*V* = 0.2 V (i.e., when ambipolar behavior is not activated), both *X*_5_ and *X*_6_ show monotonic and nearly linear responses, as shown in Figure S14c and d. In such cases, the system evolves linearly with respect to its input and previous state, which may be useful for linear memory retention but insufficient for the nonlinear transformation capabilities required in complex tasks. Figure S15 summarizes the dependence of IPC and the effective reservoir size *N*_eff_ (a dimensionality metric based on principal component analysis; see Methods for details) on Δ*V*. Both IPC and *N*_eff_ increase with a larger Δ*V*, indicating an enhanced ambipolar contribution. Notably, the third- and higher-order nonlinear capacities show a strong dependence on Δ*V*, directly demonstrating that the ambipolar behavior of graphene significantly contributes to the realization of high-order nonlinear transformations in information processing.

### Performance Evaluation of the IGR Using the NARMA10 Task

We next evaluated the IGR’s performance on the NARMA10 task, a more challenging benchmark.^15,16,21–29^ As shown in eq 4, the NARMA10 model is a dynamical system with a 10-step delay, requiring substantial MC for accurate reproduction:

4 $$y\left(k+1\right)=0.3y\left(k\right)+0.05y\left(k\right)\overset{9}{\underset{i=0}{\sum }}y\left(k-i\right)+1.5u\left(k\right)u\left(k-9\right)+0.1$$

Using the scheme in Figure 4a, we set *d*_in_ = 2. Input *u*(*k*) was applied to the gate, while delayed inputs *u*(*k* – 2) through *u*(*k* – 10) were applied to drains 1 through 5. The input data set *u*(*k*) was the same as for the NARMA2 task.

Figure 6a shows the dependence of IPC and NMSE (test phase) on *T* for the NARMA10 task. Unlike the NARMA2 task, no significant correlation was observed between *C*_tot_ and NMSE. Interestingly, NMSE reached a minimum in a high-speed region (*T* = 5 μs). A strong correlation was observed between NMSE and *C*_1_, while no significant correlation was found with nonlinear capacity, as shown in Figure 6b and c. This suggests that *C*_1_ dominates the performance in the NARMA10 task, explaining why high performance was achieved in the high-speed region where *C*_1_ is maximized. *C*_1_ of the IGR, ranging from approximately 9 to 13, is not particularly large, suggesting that the performance in this region is sensitive to *C*_1_. In scenarios where *C*_1_ is sufficiently high, higher-order capacity, as seen in the NARMA2 task, is likely to enhance the performance further. Figure 6d shows the reservoir output (red) and target (gray) under the optimal conditions, with NMSEs of 0.123 (training) and 0.124 (testing), indicating low errors. Figure 6e compares NMSEs across the PRCs. The NARMA10 task typically demands high MC, often achieved by larger PRCs with feedback circuits, such as photonic-RCs or analog circuits. Despite this, our IGR—a compact, integrable electric device—achieved top-level accuracy for this task.

**Figure 6:**
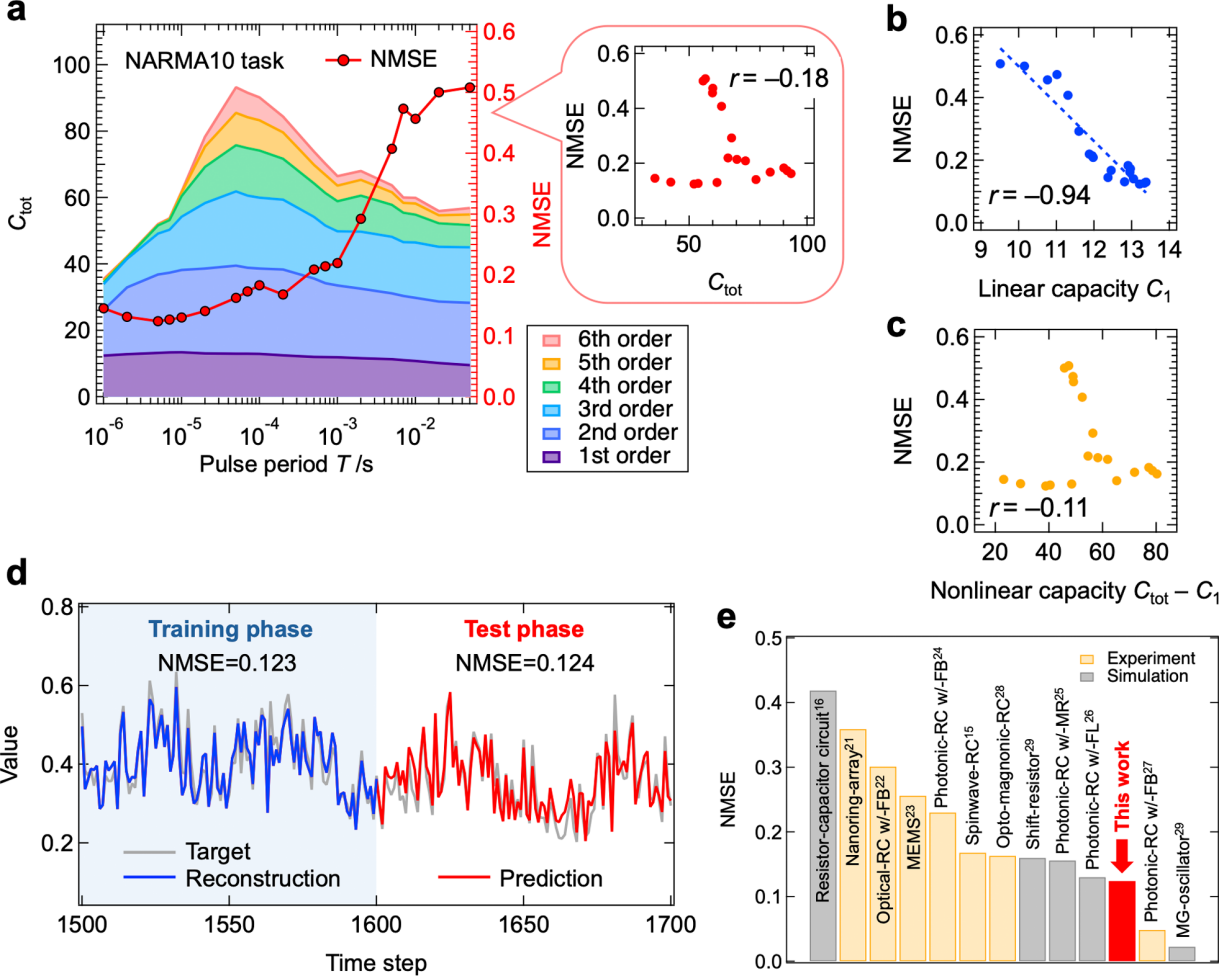
NARMA10 task performed by the IGR. **a**, Dependence of IPC (left axis) and NMSE during the test phase (right axis) on the pulse period. The inset shows a scatter plot of total capacity and NMSE. Scatter plot of NMSE versus **b**, linear capacity, and **c**, nonlinear capacity. **d**, Prediction output (red line) and target (gray line) during the training and test phases for the IGR operated under optimal conditions (*T* = 5 μs). **e**, Comparison of NMSE during the test phase with other physical reservoirs.^15,16,21–29^ MG, MR, FL, and FB refer to Mackey–Glass, microring resonator, fiber loop, and feedback, respectively.

### High Computational Performance and Efficiency of Ion-Gel/Graphene-IGR in Chaotic Time Series Prediction

We evaluated the information processing performance of the IGR with the prediction task of chaotic time series generated by the Mackey–Glass (MG) equation:

5 $$\frac{dy\left(t\right)}{dt}=\frac{0.2y\left(t-17\right)}{1+y^{10}\left(t-17\right)}-0.1y\left(t\right)$$

This task becomes increasingly difficult as the prediction horizon extends, requiring reservoirs to exhibit both nonlinearity and memory to reproduce the dynamics accurately. The widely used 1-step-ahead prediction serves as a standard PRC benchmark,^25,30–34,85^ while the more challenging 10-step-ahead task provides a higher level of difficulty for evaluating simulation-based ML models.^35,36,86–88^ For details on the generation of the chaotic time series based on the MG equation used in the task, refer to the Methods section.

Figure 7a compares the target chaotic waveform and the IGR’s prediction under optimal conditions (*T* = 70 μs) for the 1-step-ahead task. The predictions closely match the target during both training and testing phases, achieving NMSEs of 4.44 × 10^–5^ and 4.63 × 10^–5^, respectively. The phase-space plot (inset, Figure 7a) shows that the attractor formed by the reservoir output nearly overlaps with the target, demonstrating the IGR’s ability to learn and replicate the complex dynamics of chaotic systems with high accuracy and robustness. Figure 7b shows the dependence of *C*_tot_ (same as Figure 6a) and NMSE on *T* for the 1-step-ahead task. As shown in the inset, a strong correlation between *C*_tot_ and NMSE is evident, with the exceptionally high *C*_tot_ value of up to 92 (for example, 5.6 for the spin torque oscillator^84^ and around 8 for optoelectronic circuits^89^) underpinning the IGR’s outstanding performance.

**Figure 7:**
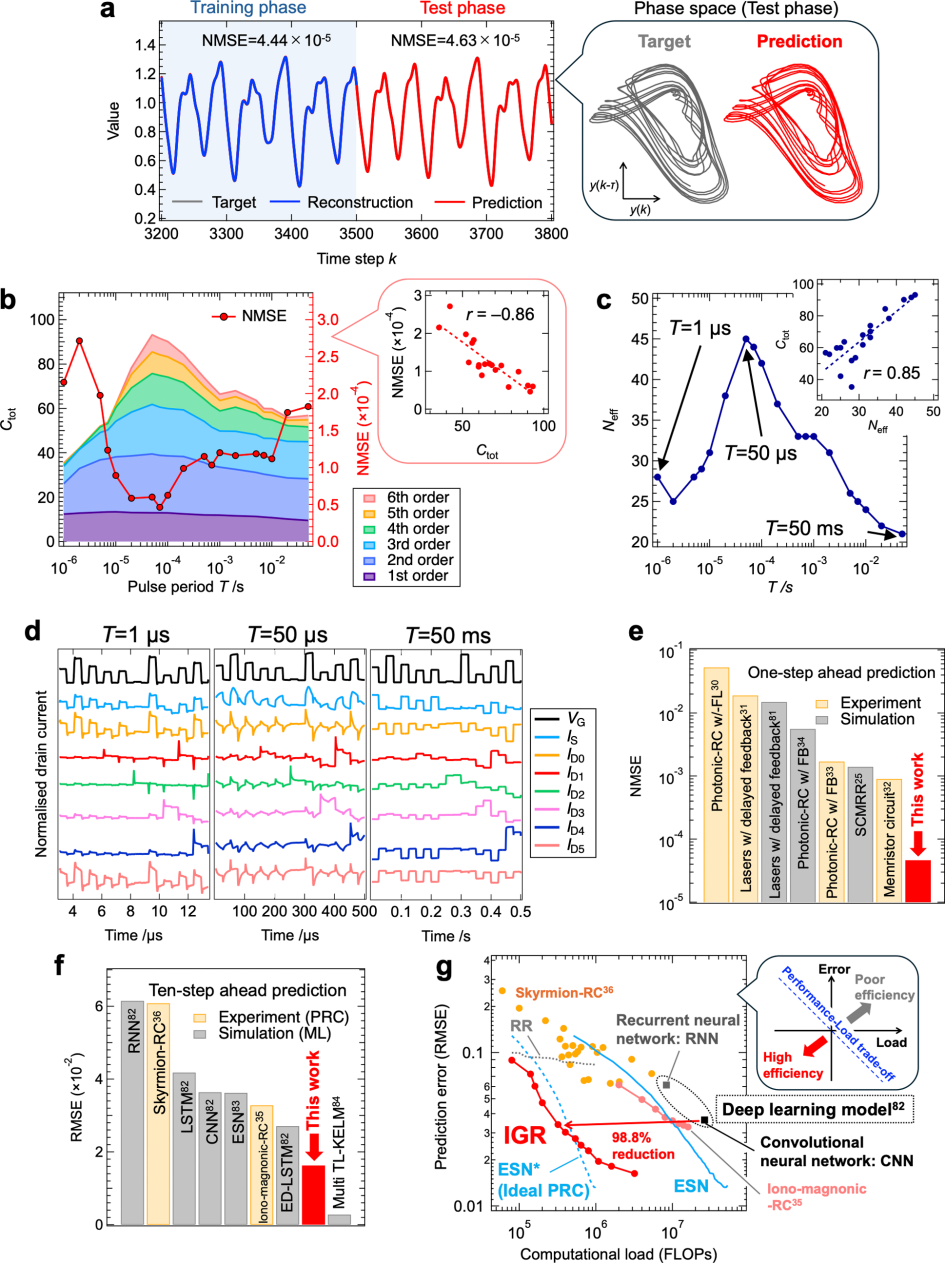
Prediction task for chaotic time series generated by the Mackey–Glass equation using the IGR. **a**, Prediction output (red line) and target (gray line) during the training and test phases for the 1-step-ahead prediction task with the IGR operated under optimal conditions (*T* = 70 μs). The inset shows the attractor in the phase space constructed by *y*(*k*) and *y*(*k*–17). **b**, Dependence of IPC (left axis) and NMSE during the test phase (right axis) on the pulse period for the 1-step-ahead prediction task. The inset shows a scatter plot of total capacity and NMSE. **c**, Dependence of effective reservoir size on the pulse period. The inset shows a scatter plot of the effective reservoir size versus total capacity. **d**, Example of current responses of the device under specific *T* conditions. **e**, Comparison of NMSE during the test phase for the 1-step-ahead prediction task with other physical reservoirs.^25,30–34,85^ SCMRR refers to a series-coupled microring resonator. **f**, Comparison of RMSE during the test phase for the 10-step-ahead prediction task with other physical reservoirs and simulation-based ML models.^35,36,86–88^ RNN refers to recurrent neural networks;^86^ CNN refers to convolutional neural networks;^86^ ED-LSTM refers to encoder-decoder LSTM;^86^ Multi TL-KELM refers to multitask learning algorithm with kernel extreme learning machine.^88^ **g**, Relationship between computational load (FLOPs) and RMSE for inference on 10,000 data points in the 10-step-ahead prediction task for the MG equation. The gray dashed line indicates the result of a simple ridge regression (RR) based on delayed inputs where the regularization parameter is set equal to that used in the IGR readout. The inset schematically illustrates the trade-off between computational load and performance for different models.

In simulated RCs, such as Echo State Networks (ESNs), *C*_tot_ is directly linked to the reservoir size *N*, constrained by (*C*_tot_ ≤ *N*).^83^ In PRCs, dimensionality is enhanced through time multiplexing; however, insufficient temporal evolution can lead to virtual nodes behaving similarly, reducing the effective reservoir size (*N*_eff_). This explains why many PRCs exhibit smaller MCs than their *N* would suggest. To assess dimensionality, principal component analysis (PCA) was performed on reservoir states in response to random inputs.^90–92^ *N*_eff_, defined as the number of principal components required to explain the original reservoir states, shows a trend similar to *C*_tot_, peaking at 45 at (*T* = 50 μs) (Figure 7c). The strong correlation between *C*_tot_ and *N*_eff_ confirms that higher dimensionality drives an increased IPC and computational performance. Figure 7d shows current responses from the IGR for representative *T* values (1 μs, 50 μs, and 50 ms). At *T* = 1 μs, the current responses are predominantly box-like, reflecting the simple charging and discharging of the EDL, with little diversity or relaxation. As *T* increases to 50 ms, all of the relaxation processes involved are fully completed, and the system’s responses once again become uniform, reducing the effectiveness of time multiplexing and diminishing *N*_eff_. At intermediate *T* = 50 μs, however, the system exhibits highly diverse current responses. The influence of both *V*_G_ and delayed inputs from the drain voltages (*V*_D1_ to *V*_D5_) results in complex interactions. This is achieved due to the high ionic conductivity (∼6.1 mS/cm) of the ion-gel, which enables delayed inputs to act as side gates, further enhancing spatial diversity. Incomplete relaxation between pulse events also contributes to effective time multiplexing, increasing dimensionality.^29,93^ The combination of diverse spatial and temporal dynamics maximizes the dimensionality and IPC, resulting in superior computational performance.

Figure 7e shows the IGR achieved top performance in the 1-step-ahead prediction task, outperforming other PRCs with much lower NMSEs than high-performance integrated-memristor circuits.^32^ For the more challenging 10-step-ahead prediction task, the IGR achieved a testing RMSE of 1.63 × 10^–2^, comparable to simulation-based ML models (Figure 7f), which typically surpass PRCs in such tasks.^35,36,86–88^ To evaluate efficiency, the computational load for inferring 10,000 data points was assessed in FLOPs (the number of floating-point operations).^94–98^ Although such computational cost analyses are not commonly conducted in the field of PRC, the number of FLOPs required for inference serves as an indicator of the machine power—and thus energy consumption—needed to drive the system, making it a standard metric in the ML community.^94–98^ In PRC, high-dimensional nonlinear mapping is physically realized by the reservoir itself, eliminating the need for FLOPs associated with this step in simulation-based RC systems. Consequently, the computational cost appears to be comparable to that of a standard ridge regression (RR); however, the actual computational capacity achieved is significantly higher due to the nonlinear transformation inherently performed by the physical reservoir. Figure 7g compares FLOPs and RMSE values across systems. The IGR, shown as red markers, outperforms RR (gray dashed line) by a substantial margin, highlighting its nonlinear, high-dimensional mapping capability—something not achievable with a simple linear system. Here, RR represents the output obtained via a linear combination of delayed inputs (i.e., *y*(*k*) = ∑_*i* = 0_^*N*_RR_^ *w*_*i*_ *u*(*k* – *i*), (*N*_RR_ = 4, 5, ..., 50)). Notably, IGR not only surpasses RR but also achieves performance on par with CNN-based models, with just 1/100 of the computational load. The blue line in the figure represents the results of a well-tuned ESN, which generally performs far better than PRCs (for ESN tuning details, see Note S6 and Figure S16). The blue dashed line represents the FLOPs for the readout network of this ESN. This can be interpreted as a benchmark for an ideal PRC system with performance equivalent to the well-tuned ESN. Notably, the IGR not only achieved efficiency comparable to that of this ideal PRC but in some cases surpassed it. In ESNs, achieving DL-level accuracy typically involves increasing reservoir size, which significantly raises computational costs (as shown in Figure 7g, the difference in efficiency between the DL models and ESN is not very large).^99^ In contrast, the IGR achieves high performance without a drastic increase in computational load, highlighting its exceptional efficiency and suitability for resource-constrained environments, such as edge AI devices.

In addition to its low computational load, the IGR’s ultrawideband temporal response allows it to respond to input signals across multiple time scales. This characteristic suggests that the device can potentially extract diverse frequency-domain features directly from its physical dynamics without relying on explicit preprocessing such as Fourier transforms. By covering a wide range of temporal components within a time-series input, the IGR may contribute to simplifying signal processing pipelines in real-time AI implementations. Furthermore, by employing time multiplexing to generate virtual nodes, the IGR constructs a high-dimensional reservoir state from a compact set of physical terminals. This dynamic use of temporal diversity enhances the system’s representational capacity and enables the construction of large-scale computational networks without increasing the physical device footprint. In this way, scalability is achieved not only through physical replication but also through temporal expansion, offering an efficient and compact platform for real-time, low-power neuromorphic computing. From the perspective of practical deployment, several factors—including scalability, long-term stability, and environmental sensitivity—must be considered when implementing IGRs in neuromorphic systems. In terms of scalability, the use of time multiplexing and virtual nodes enables the construction of large, expressive networks without increasing the physical device count, offering high computational density relative to the system volume. While the graphene channel is compatible with large-area fabrication, the choice of electrolyte remains critical. Employing patternable organic solid electrolytes, as discussed in recent studies on iontronic devices,^70–72^ is expected to improve integration and scalability. Further enhancements may also be achieved by adopting vertical device architectures. Regarding stability, graphene’s chemical inertness ensures long-term reliability, and the ambipolar transport properties remain effective as long as the electrolyte interface is preserved. Minor changes in device characteristics can be addressed through small-scale retraining of the output layer. Although the system is inherently sensitive to environmental factors, the sensitivity can be mitigated by encapsulation. Alternatively, this sensitivity may be advantageously utilized to incorporate ambient stimuli—such as light, chemical vapors, mechanical vibrations, or biological signals^100^—into the reservoir dynamics, potentially enabling environment-adaptive or multimodal computing, as envisioned in recent iontronic synapse designs.^79^ Beyond the current graphene-based configuration, further design possibilities emerge when considering alternative channel materials. For example, when extending ion-gating reservoir systems to organic mixed ionic–electronic conductors (OMIECs)^69,74–78,100^ or metal–organic frameworks (MOFs)^101^—both of which are basically nonion-blocking—the carrier modulation is achieved not through the EDL effect but via electrochemical doping. In such systems, the operational speed is governed not only by the ionic conductivity of the electrolyte but also by the ion transport properties within the channel and the strength of ion–electron coupling, as reported in recent studies.^100^ Therefore, tuning the composition of the electrolyte gel enables modulation of the response dynamics based on the gel’s ionic conductivity. Furthermore, in formulations containing multiple types of mobile ions, differences in their interactions with the channel material and their respective mobilities can lead to a composition-dependent variation in the electrochemical doping kinetics, thereby further influencing the dynamic response of the device.

## Conclusions

In this study, we demonstrated a high-performance IGR system based on an ion-gel/graphene EDLT. Our device exhibited an ultrabroadband responsive range, including sub-μs ultrafast response times, significantly overcoming the speed limitations of conventional IGRs and achieving exceptional computational performance in RC tasks. By leveraging the nonlinear dynamics, including ambipolar behavior of graphene channels and complex interactions between multiple relaxation processes, the IGR demonstrated a high IPC, far surpassing those observed in state-of-the-art PRCs, underscoring its versatility in computational applications. PCA revealed that the high *C*_tot_ arise from the diverse spatiotemporal state evolution within the IGR. In benchmark tasks such as NARMA2 and NARMA10, the IGR achieved top-level computational performance across a wide response range. Additionally, its evaluation on the Mackey–Glass chaotic time series prediction task confirmed capabilities comparable to those of simulation-based ML models, achieving superior accuracy with significantly lower computational load. The IGR also outperformed DLs in computational efficiency, offering a promising solution for power- and resource- constrained edge applications. Furthermore, this device, fabricated using graphene and ion-gel, is highly compatible with flexible electronics, expected to be the next generation of edge devices.^102^ Its ultrawideband responsiveness and ability to achieve DL-level accuracy with orders of magnitude lower computational requirements make it ideal for deployment in resource-constrained environments such as edge computing and AI devices.

## Methods

### Device Fabrication

Monolayer graphene grown by chemical vapor deposition and transferred onto a SiO_2_/Si substrate (300 nm thick SiO_2_ layer) was purchased from Graphenea (Spain). The graphene was patterned by dry etching to form channels with a width of 30 μm and lengths of 5 μm, 20 μm, and 100 μm (ch0–ch2), as well as channels with a width of 80 μm and the same lengths (ch3–ch5). These configurations are shown in the inset of Figure 1b. Raman spectroscopy of the graphene (Figure S17) confirmed its monolayer structure through the characteristic G band and strong 2D band. Drain and source electrodes were fabricated using photolithography and electron beam deposition, with Cr/Au thin films (10 and 50 nm thickness, respectively). A 500-μm-thick ion-gel electrolyte was placed on the graphene channels, and a 1-μm-thick Au foil was used as the common gate electrode.

The ion-gel was synthesized by chemically gelling the ionic liquid EMIm-TFSI with a polymer and a cross-linker (Kanto Chemical, Japan). First, EMIm-TFSI (1 mL) and a polymer solution of poly­(dimethylaminoethyl methacrylate) (PDMEMA) in toluene (200 μL) were stirred, followed by the addition of the cross-linker *N*,*N*,*N*′,*N*′-tetra­(trifluoromethanesulfonyl)-dodecane-1,12-diamine (C12TFSA) (200 μL), with all steps performed at 300 rpm for 30 min. The resulting mixture was dropped onto a Si wafer and heated at 100 °C for 15 min to induce gelation. The gel was then cut to size, transferred to the device, and dried overnight in a vacuum chamber evacuated by using a turbomolecular pump.

### Device Characterization

The transport characteristics of the device shown in Figure 1c and d were measured using a semiconductor parameter analyzer 4200A-SCS (Keithley, USA) with its source measurement unit (SMU). In the pulse *V*_G_ sweep shown in Figure 1c, *V*_G_ was applied as a pulsed voltage from −50 mV to +1.8 V in 50 mV steps and then returned to −50 mV in the same steps. The pulse width and interval were set to 10 ms and 1 s, respectively, with *V*_G_ set to 0 V during the interval. *V*_D_ was also applied as a pulse signal with the same timing as *V*_G_, set to a constant of +100 mV during the pulse and 0 V during the interval. In the DC *V*_G_ sweep measurement shown in Figure 1d, *V*_D_ was fixed at 0.5 V, while *V*_G_ was swept from 0 to 1.8 V and back to 0 V at a sweep rate of 18.5 mV/s. All measurements were conducted at room temperature in a vacuum chamber, and electrical contact to the device was achieved by using probers.

The Hall measurements shown in Figure 1e were performed using the SMU of the 4200A-SCS to evaluate changes in the mobility and carrier density of the graphene channel under ion gating. For this purpose, a Hall bar-type graphene channel device (Figure S4) was fabricated with an ion gel and Au foil placed on the channel. The device had a channel width of 30 μm, a channel length of 200 μm between the current terminals (I+ and I−), and a channel length of 20 μm between the voltage terminals (V+ and V−), matching the configuration of ch1 in Figure 1b. To cancel undesirable offset in the measured Hall voltage (*V*_H_), alternating current pulses of +10 and −10 μA were applied across the current terminals, and *V*_H_ was measured using the V+ and VH electrodes in delta-mode. The Hall coefficient was calculated from the slope of the relationship between the measured *V*_H_ and the applied magnetic field (0–0.3 T). All measurements were conducted at room temperature in a vacuum cryostat with electrical contact achieved via Al wire bonding. A dipole-type electromagnet (Tesla, Japan) was used to apply the magnetic field during the Hall measurements.

Pulse input-output characteristics for relaxation time evaluation, shown in Figure 2, were measured using the pulse measure unit (PMU) of the 4200A-SCS. With *V*_D_ fixed at 0.5 V, *V*_G_ pulses of 200 μs width were applied, and the corresponding *I*_D_ responses were recorded at a 50 MS/s sampling rate. The rise and fall times of the *V*_G_ pulses were set to 20 ns. To improve the signal-to-noise ratio of *I*_D_ and accurately evaluate relaxation times, each pulse measurement was repeated 10 times, and the averaged waveforms are shown in Figure 2. All measurements were conducted at room temperature in a vacuum chamber, with electrical contact established by using probers.

### Electrical Measurements for Information Processing Tasks

The electrical measurements for the information processing tasks discussed in Figures 4–7 were conducted using PMU of the 4200A-SCS. However, unlike the measurements for relaxation time evaluation, a single measurement was used for task execution without averaging over 10 measurements. Input information *u*(*k*) was converted into a pulsed *V*_G_ signal with pulse intensities ranging from 0 to 1.8 V and a base voltage of 1.8 V, which was applied to the gate terminal of the device. The pulse signal had a duty cycle of 50%, and the pulse period *T* was set between 1 μs and 50 ms. A constant drain voltage *V*_D0_ of 0.5 V was applied to drain terminal 0, while delayed inputs *u*(*k* – *i* × *d*_in_) (for *i* = 1, 2, ..., 5) were converted into step-like voltage signals *V*_D*i*_ with intensities ranging from 0 to 1 V and applied to the corresponding drain terminals, as shown in Figure 4a. The step signal had a period equal to that of *T*. Here, *d*_in_ represents the input delay factor, which was set to 1 for the NARMA2 task and 2 for both the NARMA10 task and the MG prediction task. Using the resulting current responses (six *I*_D_, *I*_G_, and *I*_S_), 10 virtual nodes were extracted per discrete time step (Figure 4b), generating a total of 80 reservoir states. Additionally, another 80 reservoir states were obtained by applying inverted input signals (*u*_inv_(*k*) = *u*_max_ – *u*(*k*)) through the same scheme. This approach, known as the inverted input method, compensates for the uneven distribution of information caused by the nonlinearity of the system’s mapping function, thereby maximizing the system’s information processing capabilities. Details of the inverted input method can be found in elsewhere.^39^ Using the 160 reservoir states generated through these procedures, various information processing tasks were executed.

### Readout Weight Training Algorithm

In the information processing tasks shown in Figures 4–7, the readout weights were trained by using ridge regression to minimize the error between the reservoir output and the target. The reservoir output defined in eq 1 can also be expressed as

6 y(k)=Wx(k)

Here, ***W*** = (*b*, *w*_1_,*w*_2_,···,*w*_*N*_) and ***x*** (*k*) = [1, *X*_1_(*k*), *X*_2_(*k*), ..., *X*_*N*_(*k*)]^T^ represent the readout weight vector and the reservoir state vector, respectively. Extending this to the entire training interval (*k* = 1, 2, ..., *T*_Train_), the reservoir output vector ***Y*** = [*y*(1), *y*(2), ..., *y*(*T*_Train_)] is expressed as

7 Y=WX

In this equation, ***X*** = [***x*** (1), ***x*** (2), ..., ***x*** (*T*_Train_)] represents the reservoir state matrix, and *T*_Train_ is the length of the training data. For the NARMA2 and NARMA10 tasks, *T*_Train_ was set to 1600, while for the MG prediction task, *T*_Train_ was set to 3500. Additionally, in all tasks, an unused input sequence of 100 steps was applied before the training interval to wash out the reservoir. The weights that minimize the cost function in ridge regression are given by

8 $$W=Y_{t}X^{T}{\left(XX^{T}+\lambda I\right)}^{-1}$$

Here, ***Y***_t_ = [*y*_t_(1), *y*_t_(2), ..., *y*_t_(*T*_Train_)] is the target output vector; λ is the regularization parameter, set to 5 × 10^–3^ for the NARMA2 task and 2 × 10^–3^ for the NARMA10 task and the MG prediction task. No structural optimization (e.g., pruning) was applied to the readout network. Instead, a fully connected linear readout, as defined by eq 7, was employed. The values of λ were determined based on the performance at the pulse period *T* that yielded the best results under standard linear regression (i.e., λ = 0), and these values were then used uniformly for all *T* in the subsequent analysis. The readout weights were stored on a personal computer and trained using a home-built Python code. It should be noted that the readout layer can also be physically implemented using programmable memristive arrays or artificial synaptic circuits.^103,104^

The normalized mean square error (NMSE) was used as the error metric, calculated from the reservoir output *y*(*k*) and target *y*_t_(*k*) as follows:

9 $$\mathrm{NMSE}=\frac{1}{T_{\mathrm{Data}}}\frac{\overset{T_{\mathrm{Data}}}{\underset{k=1}{\sum }}{\left[y_{t}\left(k\right)-y\left(k\right)\right]}^{2}}{\sigma^{2}\left[y_{t}\left(k\right)\right]}$$

Here, σ^2^ (•) denotes variance, and *T*_Data_ represents the data length. In the training phase, *T*_Data_ corresponds to *T*_Train_ as defined earlier. In the test phase, *T*_Data_ was set to 800 for both the NARMA2 and NARMA10 tasks and to 600 for the MG prediction task. For the 10-step-ahead MG prediction task, the RMSE was adopted as the error metric to compare computational performance with other systems:^35,36,86–88^

10 $$\mathrm{RMSE}=\sqrt{\frac{\overset{T_{\mathrm{Data}}}{\underset{k=1}{\sum }}{\left[y_{t}\left(k\right)-y\left(k\right)\right]}^{2}}{T_{\mathrm{Data}}}}$$

### Calculation Method for Information Processing Capacity in RC Systems

This section outlines the method for calculating IPC, which quantifies the nonlinearity and memory capacity in RC systems by evaluating the reconstruction accuracy of target data from reservoir states. The target data *y*_*m*_(*k*) are defined as an orthogonal polynomial encompassing all linear and nonlinear combinations of the input:

11 $$y_{m}\left(k\right)=\overset{D}{\underset{d=0}{\prod }}P_{n_{m,d}}\left[u\left(k-d\right)\right]$$

Here, *P*_*n*’_ represents an orthogonal polynomial of degree *n*’ (*n*’ = 1, 2, ...). Typically, Legendre polynomials are used,^83^ but in this study, polynomials generated using the Gram-Schmidt orthogonalization method were employed^84^ to minimize the influence of limited data size. The parameters *m*, *d*, and *D* denote the polynomial index, delay, and maximum delay, respectively. The input *u*(*k*) is a uniformly distributed random sequence used for the NARMA2 task (for *d*_in_ = 1) and NARMA10 task (for *d*_in_ = 2). The component-wise capacity *C*_*m*_ for a specific index *m* is calculated from the mean squared error (MSE = RMSE^2^) for reconstructing *y*_m_, as expressed in eq 11, from reservoir states *X*(*k*) obtained by feeding *u*(*k*) into the reservoir:

12 $$C_{m}=1-\frac{\mathrm{MSE}}{\left\langle y_{m}^{2}\right\rangle }$$

where $\left\langle y_{m}^{2}\right\rangle =\frac{1}{T_{\mathrm{Data}}}\overset{T_{\mathrm{Data}}}{\underset{k=1}{\sum }}y_{m}^{2}\left(k\right)$ . The total capacity *C*_tot_ is the sum of these component-wise capacities:

13 $$C_{\mathrm{tot}}=\overset{M}{\underset{m=1}{\sum }}C_{m}$$

Here, *M* is the total number of indices determined by the degree and delay combinations. Additionally, the degree-specific capacity *C*_*n*_ is calculated as the sum of capacities for targets with a total degree *n*:

14 $$C_{n}=\underset{m\left(n\right)}{\sum }C_{m}$$

where *m*(*n*) represents all indices corresponding to the degree *n* = ∑_*d* = 0_^*D*^ *n*_*m*,*d*_. Thus, *C*_tot_ can also be expressed as the sum of all *C*_*n*_ values, as described in eq 3.

In this study, a relatively short random sequence of 2400 steps was used for the IPC calculation. To address the challenges posed by the short sequence, Gram-Schmidt chaos,^105^ a set of polynomials obtained through Gram-Schmidt orthogonalization based on the input sequence, was employed:

15 $$P_{n^{\prime }}\left[u\left(k-d\right)\right]=u^{n^{\prime }}\left(k-d\right)-\overset{n-1}{\underset{i=0}{\sum }}c_{i}^{\left(n^{\prime }\right)}P_{i}\left[u\left(k-d\right)\right]$$

16 $$C_{i}^{\left(n^{\prime }\right)}=\frac{\overset{T_{\mathrm{Data}}}{\underset{k=1}{\sum }}P_{i}\left[u\left(k-d\right)\right]u^{n^{\prime }}\left(k\right)}{\overset{T_{\mathrm{Data}}}{\underset{k=1}{\sum }}P_{i}{\left[u\left(k-d\right)\right]}^{2}}$$

Note that *P*_0_ = 1. To avoid overestimating IPC due to the limited data, a surrogate method^84^ was applied. Input data were shuffled, and surrogate capacities *C*_sur,*m*_ were calculated for all indices *M*. A threshold *A*_th_, set as 1.5 times the maximum surrogate capacity (Figure S17a), was then used to filter capacities, setting *C*_*m*_ = 0 for values below this threshold, as shown in Figure S17b and c.

### Calculation Method for Effective Reservoir Size Using PCA

PCA is a technique that transforms high-dimensional data into independent variables called principal components.^90–92^ While commonly used in machine learning for dimensionality reduction, this study employs PCA to evaluate the high dimensionality of the IGR. PCA was applied to the reservoir state matrix ***X***, generated during the execution of the NARMA10 task, which consisted of reservoir states ***x*** (*k*) corresponding to the random sequence input *u*(*k*) (***X*** = [***x*** (1), ***x*** (2), ..., ***x*** (*T*_data_)]^T^). Each principal component is derived from the eigenvalue equation of the covariance matrix ***S*** of ***X***, expressed as ***SV*** = λ ***V***, where λ and ***V*** denote the eigenvalues and eigenvectors, respectively. When the eigenvalues are sorted in descending order (λ_1_, λ_2_, ..., λ_*N*‑1_), the eigenvector corresponding to the largest eigenvalue λ_1_ represents the first principal component, and the eigenvector corresponding to the *p*-th eigenvalue λ*p* represents the *p*-th principal component. The cumulative contribution ratio *r*_c_(*p*), which quantifies how well the original *N*-dimensional data (reservoir state matrix) is represented by the first *p* principal components, is calculated as follows:

17 $$r_{c}\left(p\right)=\frac{\overset{p}{\underset{i=1}{\sum }}\lambda_{i}}{\overset{N-1}{\underset{i=1}{\sum }}\lambda_{i}}$$

Figure S19 illustrates an example *r*_c_(*p*) curve for *T* = 50 μs, showing that the cumulative contribution ratio increases with the number of principal components *p*. In this study, the effective reservoir size *N*_eff_ is defined as the smallest number of principal components *p* that can represent the original reservoir state matrix with sufficient accuracy, defined by a threshold *r*_th_ (= 99.99%):

18 $$N_{\mathrm{eff}}=\min\left\{p\in Nr_{c}\left(p\right)\geq r_{\mathrm{th}}\right\}$$

Where N represents the set of natural numbers.

### Generation and Discretization of Chaotic Time Series

For evaluating reservoir performance on chaotic time series prediction, we used data generated from the Mackey–Glass equation, a standard benchmark system defined by the following delay differential equation:

19 $$\frac{dy\left(t\right)}{dt}=\beta \frac{y\left(t-\tau \right)}{1+y^{\gamma }\left(t-\tau \right)}-\alpha y\left(t\right)$$

As defined in eq 5, the parameters were set to α = 0.1, β = 0.2, γ = 10, and a time delay of τ = 17, under which the system exhibits chaotic dynamics.^86^ We numerically integrated the equation using the fourth-order Runge–Kutta method (RK4) implemented in Python, with a time step of Δ*t* = 0.1. The initial condition was set as *y*(0) = 0.1, and the delayed term *y*(*t* – τ) was handled by referencing previously computed values; during the initial phase, where past data were unavailable, a constant value was used for initialization. The total integration length was *N* = 50,000 steps, corresponding to a continuous time span of 5,000. From the resulting time series, we sampled every 10 steps (i.e., every 1.0 time unit) to obtain a discrete sequence {*y*_*k*_}_*k* = 0_^4999^. To eliminate transient effects, the initial 900 points were discarded, and the remaining 4,100 points were used for the prediction task. For clarity and consistency in figures, we redefined the index such that the first point used for training corresponds to *k* = 0. With this relative indexing, the first 3,500 points (*k* = 0 to 3499) were used as training data, and the subsequent 600 points (*k* = 3500 to 4099) were used as test data. Using this discrete sequence {*y*_*k*_}, we performed both 1-step-ahead and 10-step-ahead prediction tasks, in which the reservoir was trained to predict y_*k*+1_ or *y*_*k*+10_ from the present values of the input.

The analysis of the relationship between computational cost and performance, as discussed in Figure 7g, is based on this prediction task. Accordingly, the preparation of the task, namely, the generation of the data set, requires a certain computational cost due to the RK4-based numerical integration described above. However, since our focus here is on the computational load required for inference by each model (such as the IGR or other machine learning models), the computational cost associated with data set generation is excluded from the comparison.

## Supplementary Material



## Data Availability

The codes used in the current study and the data sets generated during and/or analyzed during the current study are available from the corresponding author on reasonable request.

## Notes

Modeling of the channel length dependence of on/off ratio; simulation of fast EDL dynamics based on the Nernst–Planck–Poisson equations; detailed procedures for calculating performance metrics; effect of gate voltage dynamic range on computational performance (PDF)
