# Supplementary material for: Two Orders of Magnitude Reduction in Computational Load Achieved by Ultrawideband Responses of an Ion-Gating Reservoir
Source: ACS Nano. 2025 Oct 14;19(42):36896–914. doi: 10.1021/acsnano.5c06174 (PMC12574214; doi:10.1021/acsnano.5c06174)
Supplement: Supplementary file 1 [file nn5c06174_si_001.pdf]

Supporting information

Two Orders of Magnitude Reduction in Computational Load Achieved by Ultra-wideband Responses of Ion-Gating Reservoir

Daiki Nishioka[1]*, Hina Kitano[2,3], Wataru Namiki[2], Satofumi Souma[4], Kazuya Terabe[2] and Takashi Tsuchiya[2,3]*

[1]International Center for Young Scientists (ICYS), National Institute for Materials Science (NIMS), 1-1 Namiki, Tsukuba, Ibaraki, 305-0044, Japan.

[2]Research Center for Materials Nanoarchitectonics (MANA), NIMS, 1-1 Namiki, Tsukuba, Ibaraki, 305-0044, Japan.

[3]Department of Applied Physics, Faculty of Advanced Engineering, Tokyo University of Science, Katsushika, Tokyo 125-8585, Japan

[4]Department of Electrical and Electronic Engineering, Kobe University, Kobe 657-8501, Japan

*Email: TSUCHIYA.Takashi@nims.go.jp, NISHIOKA.Daiki@nims.go.jp

Note S1: Effect of Channel Length on the Modulation Range of Drain Current (On/Off Ratio)

In this section, we examine how the channel length of the device affects the modulation range of the drain current, i.e., the on/off ratio. Figure S1(a) shows normalized transport characteristics obtained from the devices presented in Fig. 1c, corresponding to Drain0 through Drain2. These devices share a channel width of 30 μm and have channel lengths of 5 μm, 20 μm, and 100 μm, respectively. To focus on the on/off ratio of each channel, the horizontal axis represents the relative gate voltage with respect to the Dirac point, defined as $V_G' = V_G - V_{Dirac}$, and the vertical axis shows the normalized increase in drain current relative to the value at $V_G' = 0$ V. As a result, we observed that shorter channels tend to exhibit a suppressed increase in drain current in response to gate voltage. For instance, the on/off ratio z at a given gate voltage ($V_G' = -1.35$ V) was found to increase with the channel length L, as shown in Figure S1(b). To explain this behavior, we describe the drain current I_D using a composite resistance model, as illustrated in Figure S1(c). The total channel resistance R_{tot} is expressed as the sum of the V_G'-independent lead resistance R_L (primarily due to contact and lead electrodes) and the V_G'-dependent channel resistance R_C:

$$R_{\mathrm{tot}}(V_{\mathrm{G}}) = R_{\mathrm{L}} + R_{\mathrm{C}}(V_{\mathrm{G}}) \qquad \text{(S1)}$$

In this model, for shorter channels, R_C becomes smaller, making the contribution of R_L relatively larger. Therefore, even if R_C decreases under gate bias, the overall increase in I_D is limited as long as R_L remains dominant. Let $R_{C,off}$ and $R_{C,on}$ be the channel resistances in the off-state ($V_G' = 0$ V) and on-state ($V_G' = -1.35$ V), respectively. The channel-length dependence of the on/off ratio $z(L)$ can then be expressed as:

$$z(L) = \frac{R_{\mathrm{L}} + R_{\mathrm{C,off}}}{R_{\mathrm{L}} + R_{\mathrm{C,on}}} \qquad \text{(S2)}$$

The channel resistances can further be expressed using their respective resistivities:

$$z(L) = \frac{R_{\mathrm{L}} + \frac{\rho_{\mathrm{C,off}}}{W} L}{R_{\mathrm{L}} + \frac{\rho_{\mathrm{C,on}}}{W} L} \qquad \text{(S3)}$$

Here, $\rho_{C,off}$ and $\rho_{C,on}$ are the resistivities of the channel in the off and on states, respectively. The red line in Figure S1(b) shows the fitting result of the experimental data (gray dots) using the above model (Equation S3), indicating that the model accurately captures the channel-length dependence of the on/off ratio. Here, R_L was treated as a length-independent constant, and the parameters R_L, $\rho_{C,off}$ and $\rho_{C,on}$ were numerically determined to be 128.1 Ω, 1842 Ω, and 203.2 Ω, respectively.

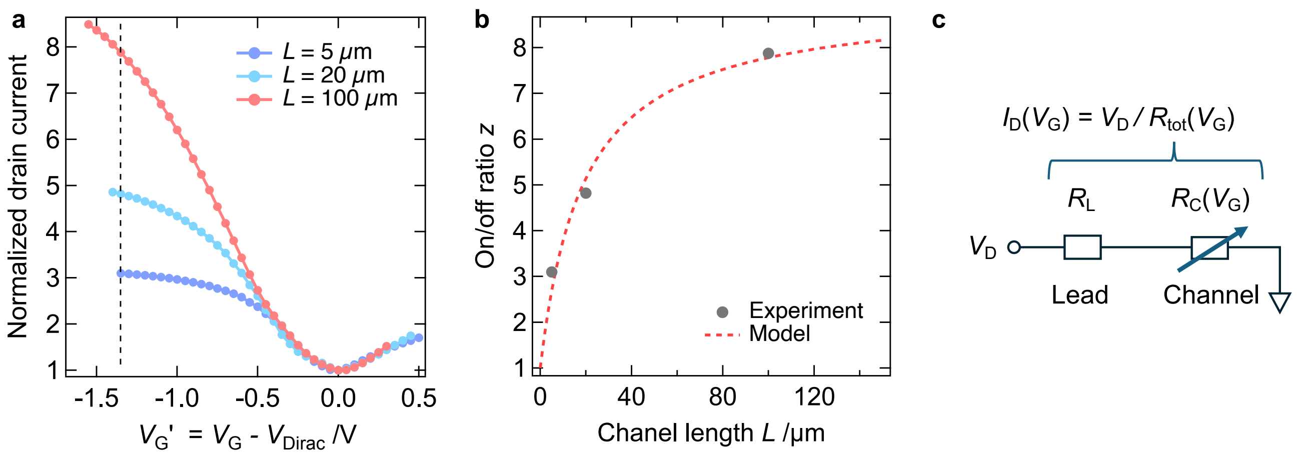

Figure S1: (a) Normalized transfer characteristics for devices with a fixed channel width of 30 µm and channel lengths of 5 µm, 20 µm, and 100 µm (Drain0–Drain2), plotted against the relative gate voltage V_G' =V_G − V_{Dirac}. The vertical axis shows the increase in drain current relative to V_G' =0 V. The dashed line indicates V_G' =-1.35 V where on/off ratios were calculated. (b) Channel-length dependence of the on/off ratio z evaluated at a fixed V_G', showing a monotonic increase with increasing channel length. The red dashed curve represents the fitting based on the composite resistance model (Eq. S3). (c) Schematic illustration of the composite resistance model, where the total channel resistance R_{tot} is composed of the gate-independent lead resistance R_L and the gate-dependent channel resistance $R_C(V_G)$.

Next, we used the obtained R_L to model the gate-voltage dependence of the drain current and validate the model. Using the composite conductance G_{tot} = 1/ R_{tot}, the drain current is expressed as:

$$I_D\,(V_G) = G_{tot}(V_G)V_D = \frac{G_L G_C(V_G)}{G_L + G_C(V_G)}\,V_D \tag{S4}$$

where G_L = 1/ R_L is the lead conductance, and G_C = 1/ R_C is the channel conductance. Ideally, $G_C = a|V_G'|$, showing linear dependence on gate voltage. However, due to the voltage drop along the channel induced by the source–drain bias V_D, the potential distribution varies within the channel. In particular, near $V_G' \sim 0$ V, both *n*-type and *p*-type regions may coexist, leading to a broadening of the conductance profile and preventing the emergence of a sharp V-shaped response. To account for this behavior, we constructed a segmented channel model in which the channel is divided into $N = L/l$ sub-regions of length l = 20 nm, as illustrated in Figure S2. Let $V_{C,i}$ be the local channel potential in the i-th segment (i=1,2,…,N). The effective gate voltage applied to that segment is $V'_{G,eff} = V_G' - V_{C,i}$, and the partial conductance G_i is defined as:

$$G_i = a \cdot |V'_{G,eff}| + b = a \cdot |V'_G - V_{C,i}| + b \tag{S5}$$

Here, $a = (W/l) \cdot \mu C_{EDL}$ is the slope of conductance change with effective gate voltage, and b is the minimum conductance at $V'_{G,eff}$ = 0 V. Due to thermally excited carriers at room temperature, $b > 0$. Assuming experimental condition of V_D = 0.5 V, the local channel potential $V_{C,i}$ was defined using a linear profile:

$$V_{C,i} = \frac{V_D}{N} \cdot \left(i - \frac{1}{2}\right) - \frac{V_D}{2} \tag{S6}$$

It should be noted that this definition ensures $V_{C,i} = 0V$ at the center of the channel ($i=N/2$), thereby preventing an artificial shift of the Dirac point caused by the introduction of V_D. This is consistent with the use of the relative gate voltage V_G', which already compensates for the Dirac point shift. The total channel conductance G_C is then given by the series combination of the segment conductances:

$$G_C = \left(\sum_{i=1}^{N} \frac{1}{G_i}\right)^{-1} = \left(\sum_{i=1}^{N} \frac{1}{a \cdot |V_G' - V_{C,i}| + b}\right)^{-1} \tag{S7}$$

Substituting into Equation S4, the drain current response becomes:

$$I_D(V_G') = \frac{G_L \cdot \left(\sum_{i=1}^{N} \frac{1}{a \cdot |V_G' - V_{C,i}| + b}\right)^{-1}}{G_L + \left(\sum_{i=1}^{N} \frac{1}{a \cdot |V_G' - V_{C,i}| + b}\right)^{-1}} V_D \tag{S8}$$

Using this model, we performed fitting to the measured data. The value of R_L was taken from the previous model (Equation S3), while a and b were treated as fitting parameters. Here, b corresponds to the minimum conductance near the Dirac point, and a is a coefficient related to the channel geometry, carrier mobility, and EDL capacitance as described above. Theoretically, based on a carrier mobility of ~3000 $cm^2/V \cdot s$ (from Hall measurements) and an EDL capacitance of ~1 $\mu F/cm^2$, the estimated value of a is approximately 4.5, which is consistent with the fitting results shown in Table S1.

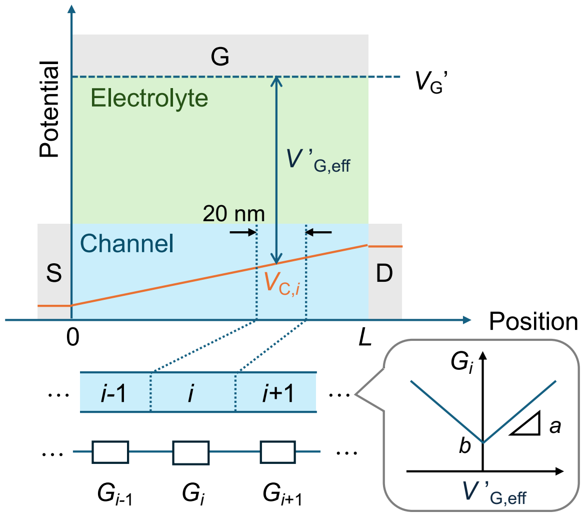

Figure S2: Segmented channel model used to describe the broadened conductance behavior near the Dirac point. The channel is divided into $N=L/l$ segments of length l=20 nm, with each segment assigned a local potential $V_{C,i}$ defined by a linear voltage drop under V_D=0.5 V. The effective gate voltage in each segment is $V_G' - V_{C,i}$, and the local conductance is given by $G_i = a|V_G' - V_{C,i}| + b$.

The red curves in Figures S3(a–c) illustrate the transport characteristics reproduced using Equation S8 and R_L = 128.1 Ω. The comparison with the experimental data (gray dots) shows that the model successfully captures the saturation behavior of I_D caused by the lead resistance. In contrast, the

simplified model without R_L (i.e., $I_D = G_C \cdot V_D$) fails to match the experimental results, particularly under large negative V_G'. This discrepancy becomes more pronounced in short-channel devices, where the contribution of R_L is relatively significant. These results strongly support the validity of our model and indicate that the observed channel-length dependence of the on/off ratio in the devices arises from the relative contribution of the lead resistance.

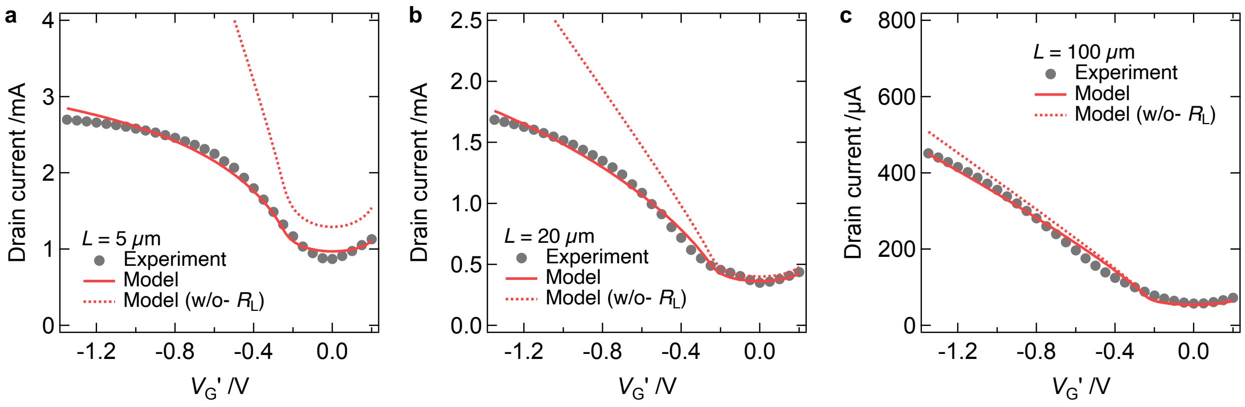

Figure S3: Comparison of the modeled (red curves) and experimental (gray dots) drain current characteristics for devices with different channel lengths of (a) 5 µm, (b) 20 µm and (c) 100 µm. The model incorporates the lead resistance R_L and the segmented channel conductance to reproduce the observed saturation behavior. Dashed curves represent the simplified model without R_L, which fails to capture the measured response, especially under large negative V_G'.

Table S1: Fitting parameters *a* and *b* for the segmented conductance model.

Channel	Parameter *a*	Parameter *b*
***L* = 5 μm**	7.54	0.135
***L* = 20 μm**	5.61	0.294
***L* = 100 μm**	3.76	0.206

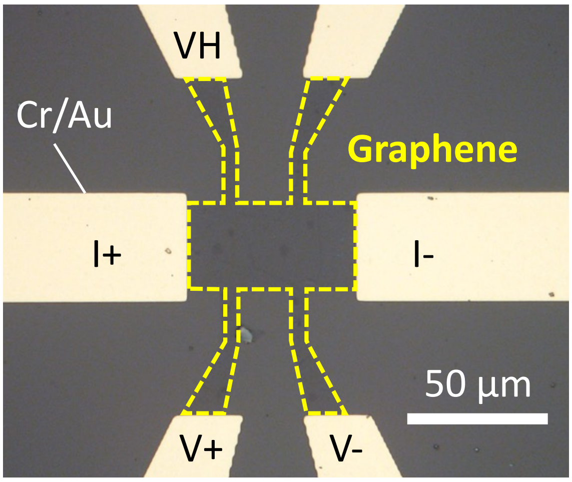

Figure S4: Optical microscope image of the Hall bar-type graphene channel. The Hall measurements shown in Figure 1e were performed using an EDLT fabricated by placing ion-gel and Au foil on this Hall bar-type channel. I+ and I- are the current terminals, V+ and V- are the voltage terminals, and VH is the terminal for Hall voltage measurements.

Note S2: Simulation-Based Reproduction and Interpretation of 100~ns Switching Behavior

The experimentally observed switching response on the order of 99 ns can be quantitatively explained by incorporating electrostatic screening effects into numerical simulations based on the Nernst-Planck-Poisson (NPP) equations as follows. In this framework, the NPP equations are solved to obtain the time evolution of ionic concentrations (both anions and cations), fluxes, and electrostatic potential within the ion gel, where the presence of the graphene channel is taken into account by calculating the charge distribution using a tight-binding (TB) method combined with a quantum capacitance model. The electrostatic potential is obtained by solving the Poisson equation over the entire simulation domain, including the graphene layer, under the boundary condition that a gate voltage is applied to the opposite side of the ion gel. Then the channel current in graphene is calculated using the Boltzmann transport theory. The detailed methodology has been reported previously[1].

In the present study, we explicitly consider the electrostatic screening within the ion gel, which acts as the gate dielectric. Given the relatively high ionic conductivity of the gel, σ = 6 mS/cm, it is reasonable to assume that the electric field inside the electrolyte is dynamically suppressed via ionic motion, which is captured by solving the time-dependent NPP equations.

That is, we consider a Debye-length-dependent additional term in the Poisson equation as

$$\frac{d^2\phi(z)}{dz^2} = -\frac{\rho(z)}{\varepsilon} + \frac{(\phi(z) - \phi_{\mathrm{bulk}})}{\lambda_{\mathrm{D}}^2} \quad \text{(S9)}$$

along the ion-gel thickness direction (normal to the graphene plane), where the second term on the right-hand side effectively accounts for the electrostatic screening arising from mobile ions under the assumption of linearized Boltzmann statistics, with the Debye screening length $\lambda_{\mathrm{D}} = \sqrt{\varepsilon k_B T / 2ne^2}$ estimated based on the ion density n. In the above Poisson's equation, ϕ_{bulk} denotes the bulk electrostatic potential determined by the charge neutrality condition, and is assumed to be moderately shifted toward the graphene channel potential near the graphene interface (within a few nanometers) due to asymmetric electrostatic coupling and incomplete ionic screening.

In the actual simulation, we assume the ion density $n = 3.23 \times 10^{27}\ \mathrm{m}^{-3}$ and the diffusion constant $D = 1.5 \times 10^{-11}\ \mathrm{m}^2/\mathrm{s}$ for both anion and cation. These parameter choices are consistent with the experimentally observed conductivity $\sigma \approx$ 6 mS/cm through the Nernst-Einstein relation: $\sigma = 2e^2 nD/k_{\mathrm{B}}T$ at the room temperature. The permittivity is assumed as $\varepsilon = 10\varepsilon_0$. Figure S5 shows the time evolution of the normalized graphene channel current in response to a pulsed gate voltage applied to a graphene FET with a 500 µm-thick ion gel as the gate dielectric. The pulse width is 5 µs, with the base (off-state) gate voltage set to V_{b} = -0.1 V and the input (on-state) gate voltage set to V_{in} = -0.55 V. As can be seen from the result, a switching response on the order of 100 ns, comparable to the experimental observation, is successfully reproduced. The agreement between the experimental and simulation results suggests that the relaxation time on the order of 100 ns can be interpreted as $\tau = d_{\mathrm{EDL}}^2/D$, where the typical width of the electric double layer is assumed to be d_{EDL} =1.2 nm and the diffusion constant is taken as $D = 1.5 \times 10^{-11}\ \mathrm{m}^2/\mathrm{s}$ as mentioned above, yielding $\tau \approx$ 100 ns.

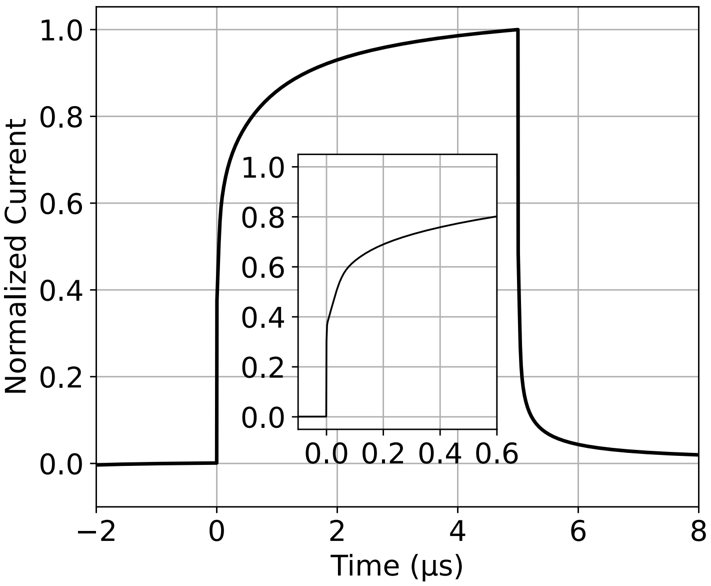

Figure S5: Time evolution of the normalized graphene channel current in response to a pulsed gate voltage applied to a graphene FET with a 500 μm-thick ion gel as the gate dielectric. The inset shows a magnified view of the time window from 0 to 0.5 μs, highlighting the fast switching response on the order of 100 ns.

Note S3: C_{EDL} Evaluation

The electric double-layer capacitance C_{EDL} of the ion-gel/graphene EDLT was 0.91 μF/cm^2 in the p-type region and 0.88 μF/cm^2 in the n-type region. These values were calculated by applying the widely used relationship $\frac{dQ}{dV_G} = C_{EDL}$ (the differential capacitance of the EDL) to the changes in Hall density and electron density obtained from Hall measurements as a function of V_G[2-4]. Figure S6 shows the carrier density dependence obtained from Hall measurements, with linear fitting performed over the p-type region (from V_G-V_{Dirac}=-1.525 V to -0.425 V) and the n-type region (from V_G-V_{Dirac}=0.375 V to 1.275 V). From these fittings, C_{EDL} was calculated, and as shown in Figure S6, the fitting lines match well across a wide V_G range. This result indicates that C_{EDL} has little dependence on the applied V_G. The C_{EDL} values calculated here are consistent with experimentally and theoretically estimated values for graphene EDLTs using ionic liquids[3-5]. The relatively low C_{EDL}<1 μF/cm^2, compared to the typical ~10 μF/cm^2 for ionic liquid-based EDLs[6], arises from the limitation imposed by the series quantum capacitance C_q[5].

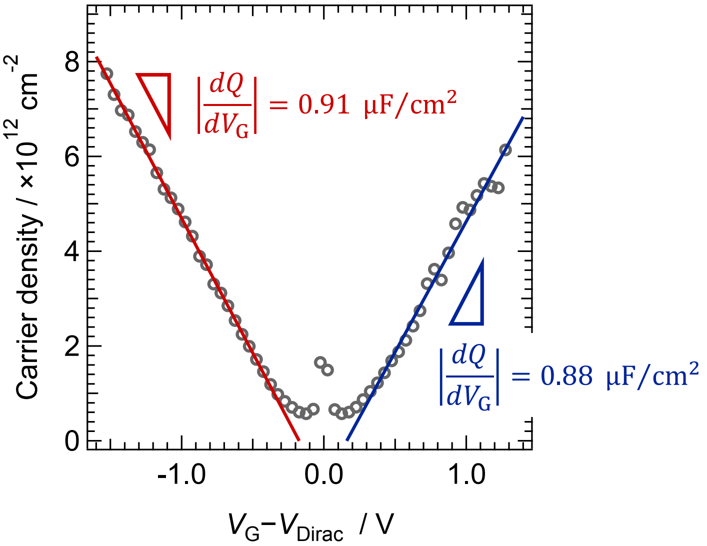

Figure S6: Carrier density changes in the graphene channel as a function of the applied gate voltage obtained from Hall measurements. The red and blue lines represent linear fittings in the p-type and n-type regions, respectively, and the slopes of these fittings were used to calculate the electric double-layer capacitance C_{EDL}.

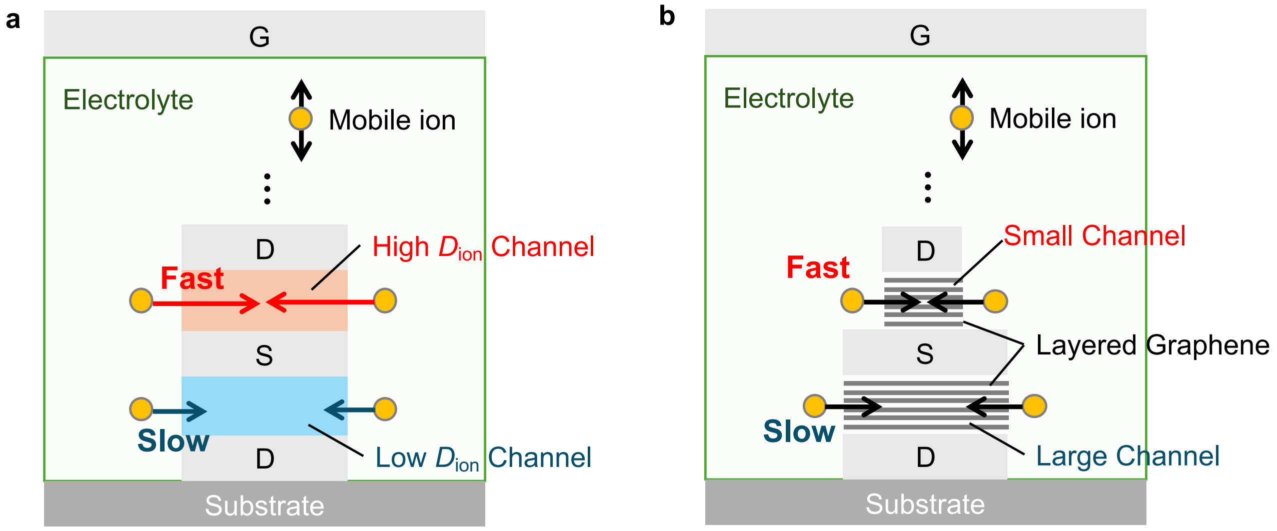

Figure S7: Conceptual design of vertically stacked ion-gating transistors with multi-timescale responses. (a) Schematic illustration of a vertical organic electrochemical transistor (vOECT) composed of stacked channel materials with different ion diffusion coefficients D_{ion}, enabling engineered temporal dynamics across layers. (b) Vertically integrated graphene-based architecture, where varying the channel area and using multilayer graphene allows integration of multiple timescales. Ion trapping in multilayers may further enhance tunability.

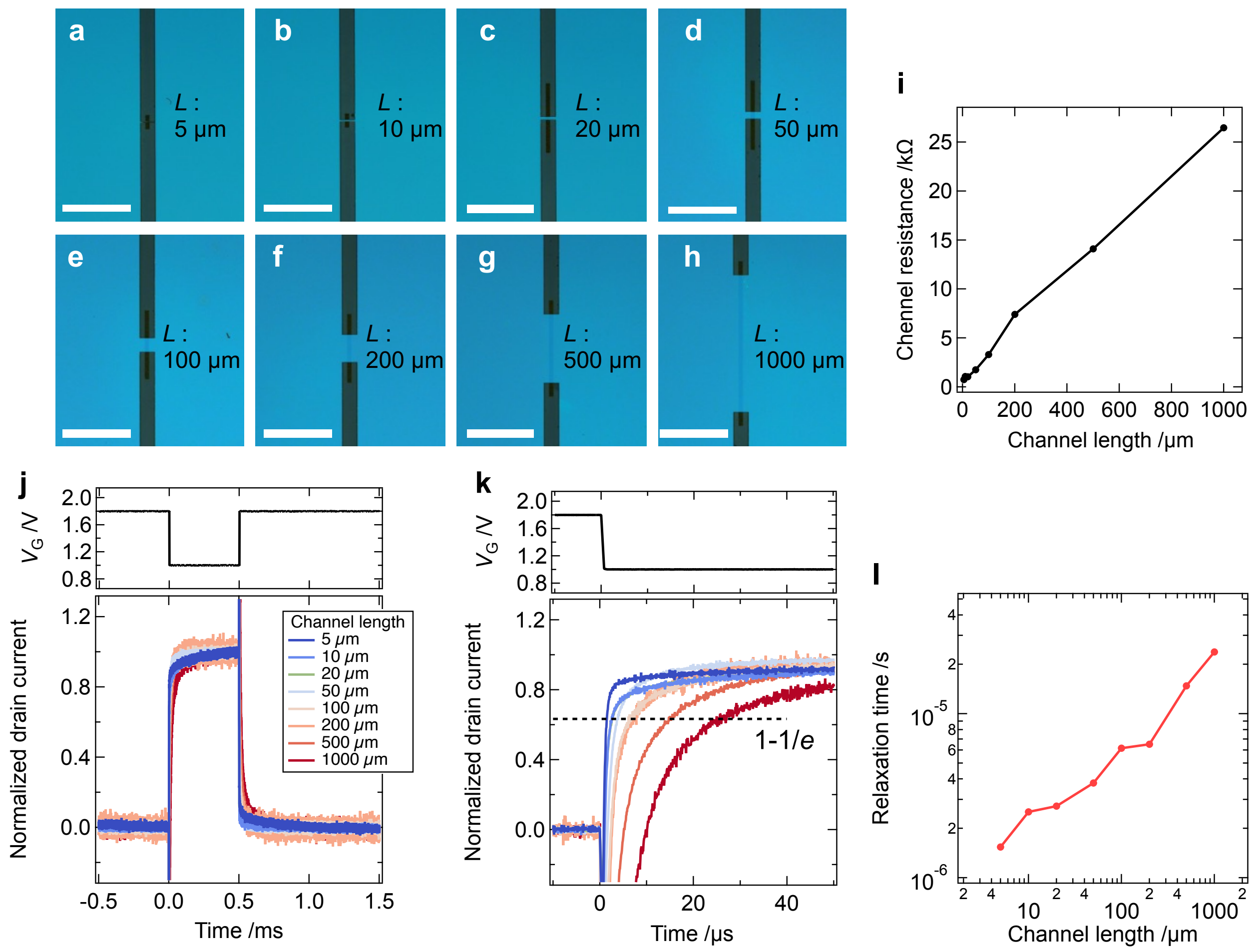

Figure S8: Influence of channel geometry on relaxation time. (a)–(h) Optical microscope images of graphene channels and source/drain electrodes for individually fabricated devices. The channel width is fixed at 30 μm, and the channel lengths L are 5 μm, 10 μm, 20 μm, 50 μm, 100 μm, 200 μm, 500 μm, and 1000 μm, respectively. The white boxes in the images indicate a scale bar of 500 μm. (i) Channel resistance of graphene as a function of channel length. (j) Overview and (k) magnified view of the normalized drain current responses to pulsed gate voltages for each device. (l) Relaxation time as a function of channel length.

Note S4: Detailed calculation method for information processing capacity

Here, we describe the detailed procedure for calculating the information processing capacity (IPC). The IPC is evaluated based on the reservoir's ability to reconstruct a target signal y_m, which is defined as follows, from a random input sequence $u(k)$:

$$y_m(k) = \prod_{d=0}^{D} P_{n_{m,d}}[u(k-d)] \tag{S10}$$

In this expression, m is the index representing a specific polynomial term; d is the delay; D is the maximum delay; and $n_{m,d}$ denotes the polynomial order associated with a given index and delay. The polynomial $P_{n'}$ represents the n'-th order orthogonal polynomial generated via the Gram-Schmidt process, and is defined as:

$$P_{n'}[u(k-d)] = u^{n'}(k-d) - \sum_{i=0}^{n-1} c_i^{(n')} P_i[u(k-d)] \tag{S11}$$

$$c_i^{(n')} = \frac{\sum_{k=1}^{T_{\mathrm{Data}}} P_i[u(k-d)]u^{n'}(k)}{\sum_{k=1}^{T_{\mathrm{Data}}} P_i[u(k-d)]^2} \tag{S12}$$

with $P_0 = 1$. For example, the first- and second-order polynomials are given by:

$$P_1[u(k-d)] = u(k-d) - \bar{u} \tag{S13}$$

$$P_2[u(k-d)] = u^2(k-d) + \frac{\bar{u}\overline{u^2} - \overline{u^3}}{\overline{u^2} - \bar{u}^2} u(k-d) + \frac{\bar{u}\overline{u^3} - \overline{u^2}^2}{\overline{u^2} - \bar{u}^2} \tag{S14}$$

where $\overline{u^{n'}} = \frac{1}{T_{\mathrm{Data}}} \sum_{k=1}^{T_{\mathrm{Data}}} u^{n'}(k)$ (n'=1,2,…). Higher-order polynomials such as P_3 and beyond can be defined recursively using the above relation.

Next, for each index m (corresponding to a specific combination of delay and polynomial order), we perform a regression task in which the reservoir is trained to reconstruct the target signal y_m defined above. Since y_m is a product of multiple polynomials of degree $n_{m,d}$, the total degree n of the target function can be written as:

$$n = \sum_{d=0}^{D} n_{m,d} \tag{S15}$$

Let $y(k)$ denote the output of the reservoir. The accuracy of this reconstruction task is evaluated based on the error between $y_m(k)$ and $y(k)$, and is defined as the component-wise capacity C_m:

$$C_m = 1 - \frac{\sum_{k=1}^{T_{\mathrm{Data}}} [y_m(k) - y(k)]^2}{\sum_{k=1}^{T_{\mathrm{Data}}} [y_m(k)]^2} \tag{S16}$$

By performing the above reconstruction task for all index values m, the overall IPC can be obtained. In addition, by focusing only on the components with a specific polynomial degree n, the sum of C_m over the corresponding indices $m(n)$ is defined as the degree-specific capacity C_n. The total IPC, C_{tot},

is then given by the sum of C_m over all indices:

$$C_n = \sum_{m \in m(n)} C_m \tag{S17}$$

$$C_{\mathrm{tot}} = \sum_{m} C_m \tag{S18}$$

As an illustrative example, we describe the step-by-step calculation of the first-order capacity C_1 under the optimal condition for the NARMA2 task (T = 100 μs). For n = 1, the target signal $y_m(k)$ is given by Eq. (S10) as:

$$\begin{aligned} y_m(k) &= P_0[u(k-0)] \times P_0[u(k-1)] \times P_0[u(k-2)] \times \ldots \\ &\times P_0\big[u\big(k-(d-1)\big)\big] \times P_1[u(k-d)] \times P_0\big[u\big(k-(d+1)\big)\big] \times \ldots \times P_0[u(k-D)] \\ &= P_1[u(k-d)] \end{aligned} \tag{S19}$$

Using Eq. (S13), the final form of the target with n = 1 can be written as:

$$y_m(k) = u(k-d) - \bar{u} \tag{S20}$$

This reconstruction task essentially examines whether the reservoir can "recall" the value of the input sequence $u(k\text{-}d)$ with a delay d. This corresponds closely to the widely known concept of memory capacity (MC) in the field of reservoir computing, though slight differences exist—such as the inclusion of d = 0 and the method used for evaluating task performance. Figures S9 (a) to (c) shows representative examples of the target $y_m(k)$ and the reservoir output $y(k)$ at various delays d = 0, 7, 10. As can be observed, the reservoir accurately reconstructs the target at small delays, whereas the reconstruction deteriorates as d increases. This is because the task becomes more difficult as the reservoir is required to retrieve older information from the input history. The dependence of the task accuracy (i.e., component-wise capacity C_m) on delay d is summarized in Figure S9 (d). The observed decrease in C_m with increasing d represents the device's "forgetting curve", indicating the extent to which the reservoir can "linearly" retrieve past information. The first-order capacity C_1, corresponding to n = 1, is then obtained by summing the partial capacities as follows:

$$C_1 = \sum_{\substack{m \in \\ m(n=1)}} C_m = \sum_{d=0}^{D} C_{m(n=1)}(d) \tag{S21}$$

To prevent underestimation of C_1, the maximum delay D was set to a sufficiently large value (e.g., D = 40). Furthermore, to avoid overestimation of the IPC, the values of C_1 shown in the main text were processed using a surrogate-based thresholding method, as described in the Methods section; specifically, any C_m below a predetermined threshold was set to zero.

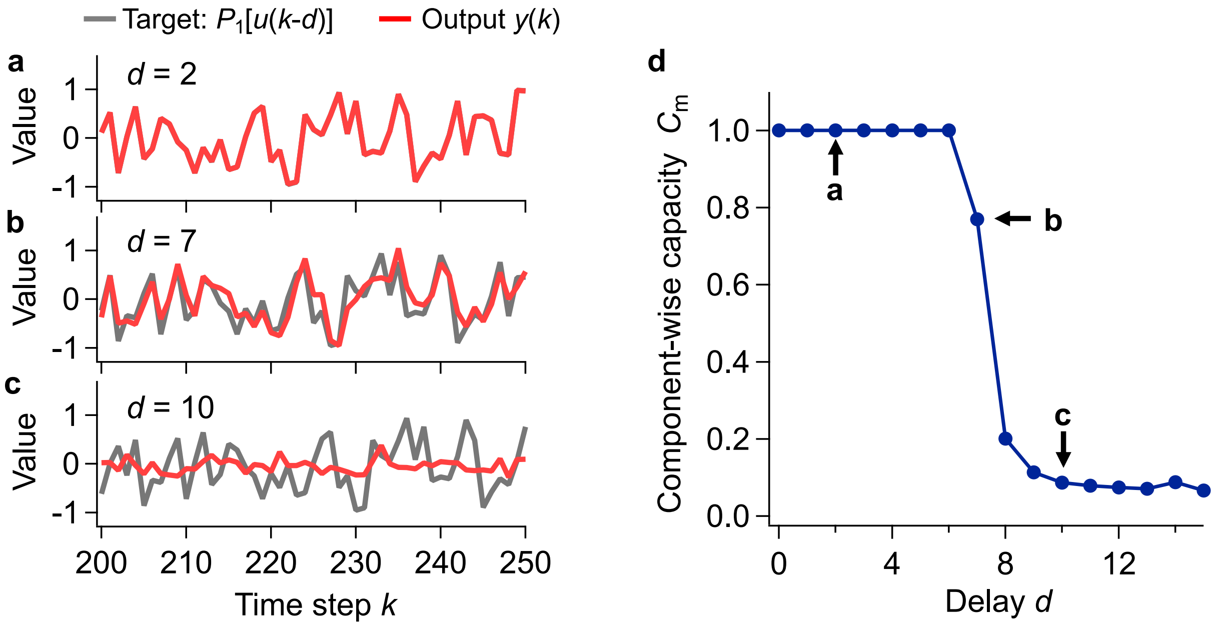

Figure S9: First-order reconstruction task. Representative examples of the target $P_1[u(k\text{-}d)]$ and reservoir output $y(k)$ for various delays (a)d=0, (b) d=7 and (c) d=10 in the first-order reconstruction task (n=1). The reservoir accurately reconstructs the target at short delays but exhibits degraded performance as the delay increases. (d) Delay dependence of the component-wise capacity C_m in the first-order task. The monotonic decay of C_m with increasing delay illustrates the forgetting curve of the reservoir and reflects its linear memory capability.

Next, we describe the procedure for calculating the second-order capacity C_2, corresponding to $n = 2$. A representative example of a second-order target y_m is given by:

$$y_m(k) = P_2[u(k-d)] = u^2(k-d) + \frac{\bar{u}\overline{u^2} - \overline{u^3}}{\overline{u^2} - \bar{u}^2} u(k-d) + \frac{\bar{u}\overline{u^3} - \overline{u^2}^2}{\overline{u^2} - \bar{u}^2} \qquad (S22)$$

As in the $n = 1$ case, a reconstruction task is conducted for each delay d, and the second-order capacity is computed based on the task accuracy. This task evaluates the reservoir's ability to reconstruct waveforms that are quadratic transformations of the input signal $u(k\text{-}d)$. Figures S10 (a) to (c) shows examples of the target $y_m(k)$ and the reservoir output y(k) for d = 0, 6, 10. Figure S10 (d) also presents the dependence of the component-wise capacity C_m on delay d. Similar to the linear case shown in Figure S9 (d), C_m decreases as d increases, reflecting the increasing difficulty of the task. However, in this task, the reservoir must possess not only memory but also nonlinear transformation capability. As a result, the decay of C_m with increasing d is noticeably faster compared to the linear (first-order) case. This trend becomes more pronounced as the polynomial degree n increases, indicating that tasks requiring both memory and nonlinearity are inherently more challenging.

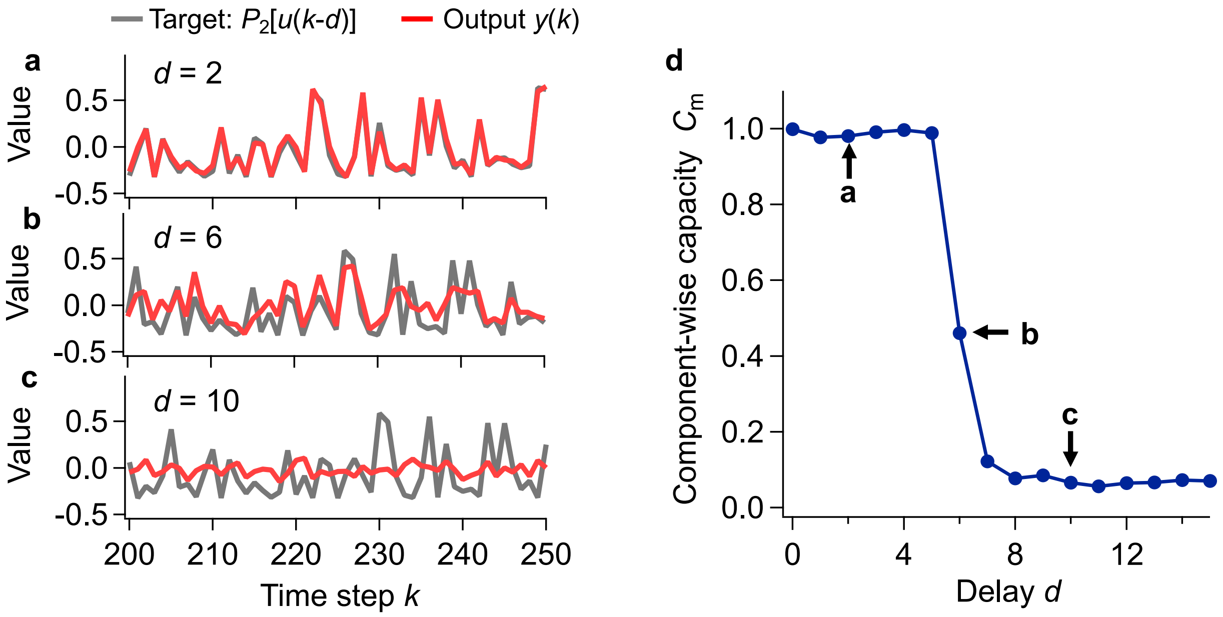

Figure S10: Second-order reconstruction task with single delay. Examples of target $P_2[u(k\text{-}d)]$ and output $y(k)$ waveforms for the second-order reconstruction task for various delays (a) d=0, (b) d=6 and (c) d=10. The reservoir performance declines with increasing delay due to the added nonlinearity requirement. (d) Delay dependence of the component-wise capacity C_m in the Second-order task.

Another class of second-order targets y_m is given by:

$$y_m(k) = P_1[u(k-d_1)] \times P_1[u(k-d_2)] = \{u(k-d_1) - \bar{u}\}\{u(k-d_2) - \bar{u}\} \qquad \text{(S23)}$$

In this case, the task involves reconstructing second-order targets generated by the product of two first-order polynomials, $P_1[u(k\text{-}d_1)]$ and $P_1[u(k\text{-}d_2)]$, each with different delays d_1 and d_2 ($d_1 < d_2$). Figure S11 summarizes the dependence of C_m on both d_1 and d_2. As in the previous case, C_m decreases as either delay increases, again reflecting the growing difficulty of reconstructing temporally distant nonlinear features. For $n = 2$, the second-order capacity C_2 is calculated as the sum of component-wise capacities from the two types of tasks described above.

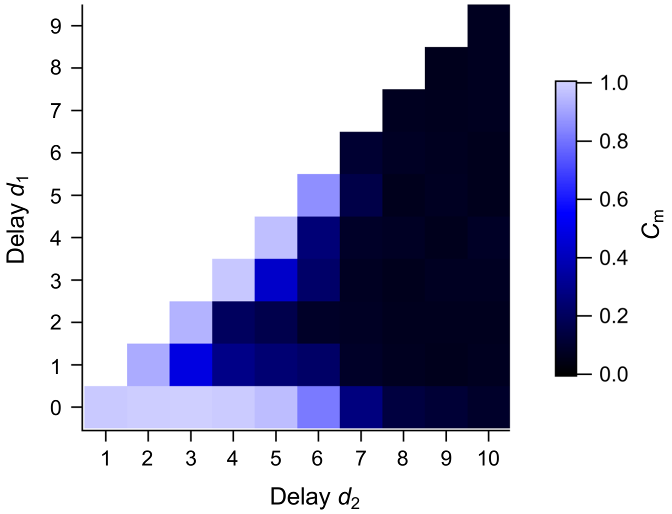

Figure S11: Second-order reconstruction task with two delays. Delay dependence of C_m in the second-order task based on $y_m(k)=P_1[u(k-d_1)]\cdot P_1[u(k-d_2)]$ ($d_1<d_2$). As either d_1 or d_2 increases, the task becomes more difficult and capacity decreases, reflecting the joint challenge of memory and nonlinear transformation.

For higher-order capacities ($n \geq 3$), the procedure is extended in a similar manner. For example, for $n = 3$, the IPC is evaluated using tasks that reconstruct targets defined by $P_3[u(k-d)]$, $P_2[u(k-d_1)]\times P_1[u(k-d_2)]$ ($d_1 \neq d_2$), and $P_1[u(k-d_1)]\times P_1[u(k-d_2)]\times P_1[u(k-d_3)]$ ($d_1 < d_2 < d_3$). By evaluating reconstruction performance for various combinations of polynomial orders and time delays, IPC provides a comprehensive measure of both the memory and nonlinear processing capability of a given reservoir. Ideally, the maximum nonlinearity order and maximum delay should be set to sufficiently large values to fully characterize the reservoir's capability. However, increasing these values leads to a combinatorial explosion in the number of reconstruction tasks. Therefore, in this study, the maximum polynomial degree was limited to six, and the maximum delay D was set individually for each n, as summarized in Table S2. Under this condition, the total number of target indices M is 3.4×10^3. Table S3 shows representative IPC values (after surrogate-based thresholding) under selected conditions.

Table S2: Maximum delay *D* values used for each polynomial degree *n* in the IPC calculation. Larger *n* corresponds to smaller maximum delays to manage computational complexity while retaining sufficient evaluation coverage.

Degree *n*	Maximum delay *D*
**1**	40
**2**	30
**3**	20
**4**	15
**5**	15
**6**	11

Table S3: Representative values of information processing capacity after surrogate-based thresholding.

Condition	C_1	C_2	C_3	C_4	C_5	C_6	C_{tot}
**T=50 μs / d_{in}=1**	8.24	19.4	23.1	16.5	12.2	9.48	88.9
**T=50 μs / d_{in}=2**	13.0	26.5	22.3	13.9	9.74	7.70	93.2

Note S5: Detailed calculation method for normalized mean squared error

Here, we describe the detailed procedure for calculating the normalized mean squared error (NMSE). NMSE was computed over all discrete time steps k, based on the squared error between the reservoir output $y(k)$ and the target signal $y_t(k)$. As defined below, NMSE is obtained by normalizing the mean squared error (MSE) with the variance of the target:

$$\mathrm{NMSE} = \frac{\mathrm{MSE}}{\sigma^2} \tag{S24}$$

$$\mathrm{MSE} = \frac{1}{T_{\mathrm{Data}}} \sum_{k=1}^{T_{\mathrm{Data}}} [y_t(k) - y(k)]^2 \tag{S25}$$

Here, T_{Data} is the data length; σ^2 denotes the variance of the target signal, and is defined as:

$$\sigma^2 = \frac{1}{T_{\mathrm{Data}}} \sum_{k=1}^{T_{\mathrm{Data}}} [y_t(k) - \bar{y}_t]^2 \tag{S26}$$

$$\bar{y}_t = \frac{1}{T_{\mathrm{Data}}} \sum_{k=1}^{T_{\mathrm{Data}}} y_t(k) \tag{S27}$$

As an illustrative example, we show the NMSE computation in the test phase of the NARMA2 task under optimal conditions (T = 100 μs). Figure S12 displays both the target $y_t(k)$ and the reservoir output $y(k)$ over the 800-step time series, with the temporal squared error $[y_t(k) - y(k)]^2$ plotted on the right vertical axis. The MSE is obtained by summing the squared error over all time steps (gray shaded area in the figure) and dividing by the number of steps (T_{Data} = 800). In this case, MSE was found to be 4.09×10^{-6}. In this study, we adopted NMSE as the error metric to enable fair comparisons with other PRC systems. Unlike raw MSE, NMSE accounts for the variability of the target signal by normalizing with its variance (Eq. S24). As described in the main text, the resulting NMSE under these conditions was 7.35×10^{-3}.

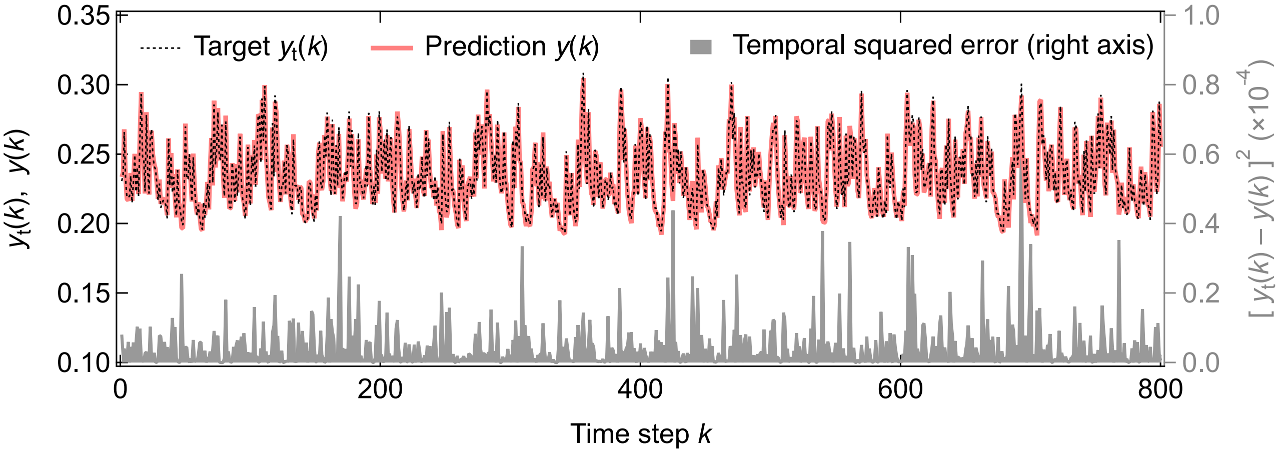

Fig S12: NMSE computation in the NARMA2 task. Target signal $y_t(k)$, reservoir output $y(k)$, and squared error $[y_t(k) - y(k)]^2$ over 800 time steps. The shaded area corresponds to the temporal squared error used to compute MSE, which is then normalized by the target variance to obtain NMSE.

It is worth noting that in the field of PRC, the term "NMSE" is sometimes used to refer to two different normalization schemes. One is the definition adopted in this study (Eq. S24), while the other normalizes MSE by the mean squared value of the target signal rather than its variance. Therefore, when comparing NMSE values across different studies, it is essential to carefully verify the underlying definition. In this work, only NMSE values defined equivalently to Eq. S24 were used for performance comparisons with other PRC implementations.

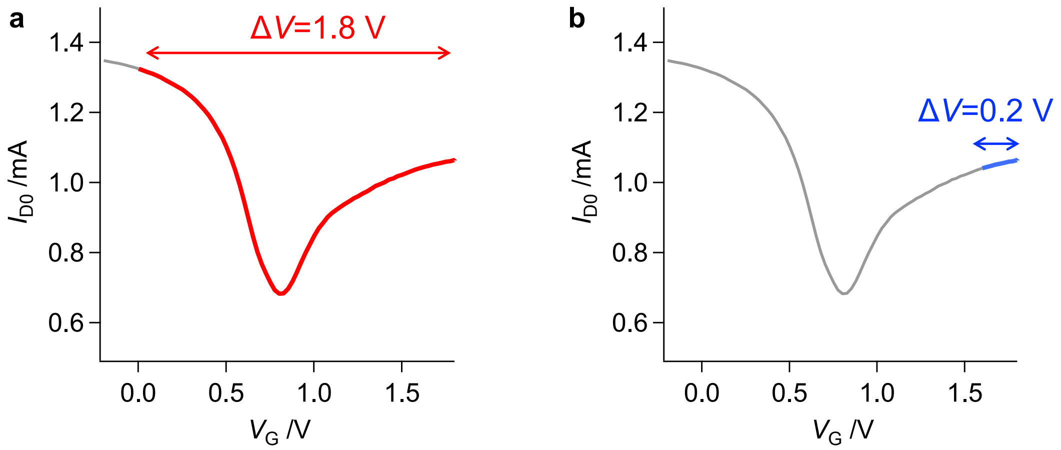

Figure S13: Gate voltage dynamic range selection for evaluating ambipolar behavior. Schematic illustration of how varying the minimum gate voltage V_{in} from 0.2 V to 1.8 V at a fixed base voltage V_b=1.8 V alters the dynamic range ΔV=|$V_b - V_{in}$| of the gate pulse. This range effectively selects the operating region of the device's transfer characteristics, thereby enabling control over the contribution of ambipolar transport in information processing.

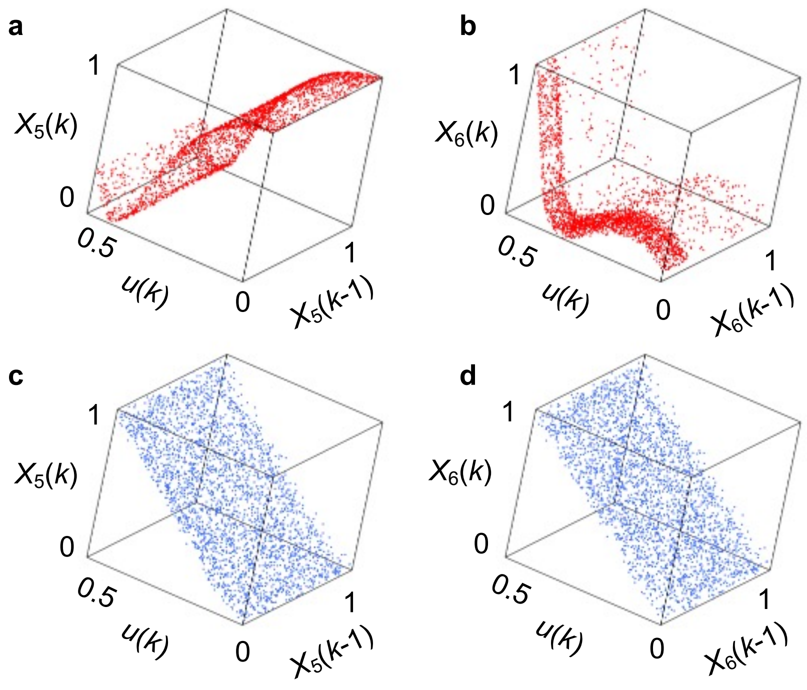

Figure S14: Influence of ambipolar transport on the reservoir's mapping function. 3D plots of reservoir states as a function of current input $u(k)$ and past state $X(k$-1). (a, b) Under ΔV=1.8 V, X_5 and X_6 exhibit distinct nonlinear surfaces, reflecting the contribution of ambipolar behavior. (c, d) Under ΔV=0.2 V, both states show linear, monotonic behavior, indicating suppressed ambipolarity. These results demonstrate that ambipolar transport enhances the diversity and nonlinearity of the system's mapping functions.

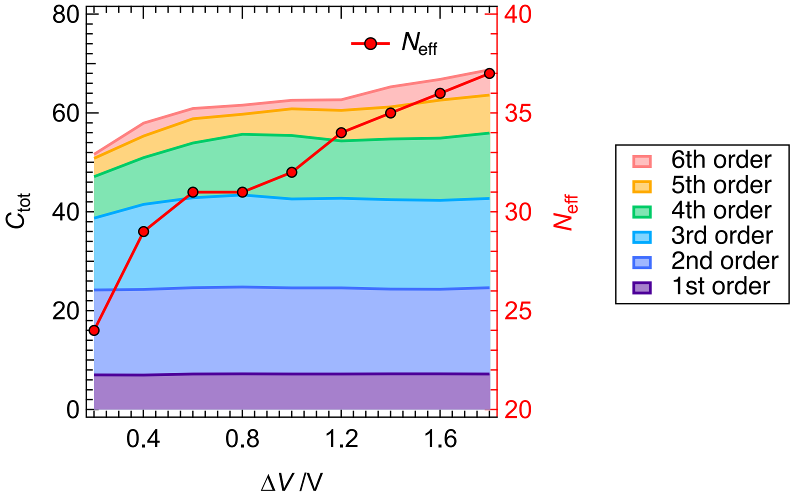

Figure S15: Dependence of IPC and effective reservoir size on the gate voltage dynamic range ΔV. Both the total information processing capacity and the effective reservoir size N_{eff} (calculated via principal component analysis) increase with ΔV. In particular, higher-order nonlinear capacities ($n \geq 3$) show strong dependence on ΔV, indicating that the ambipolar behavior of graphene significantly contributes to the realization of nonlinear transformations in reservoir computing.

Note S6: ESN Setup for the Mackey-Glass Prediction Task

To evaluate the efficiency of the developed PRC, we compared it with a well-tuned Echo State Network (ESN) in the 10-step-ahead prediction task for time series generated by the Mackey-Glass (MG) equation, as shown in Figure 7g. This section describes the setup and optimization method of the ESN. In this study, we adopted a standard ESN model[7)], where the state evolution is described by the following equation (S28):

$$\boldsymbol{x}(k) = \boldsymbol{f}[\boldsymbol{W}_{\mathbf{rec}}\boldsymbol{x}(k-1) + \boldsymbol{W}_{\mathbf{in}}\boldsymbol{u}(k)] \tag{S28}$$

Here, $\boldsymbol{x}$ is the reservoir state vector, $\boldsymbol{f}$ is the mapping function, $\boldsymbol{W}_{\mathbf{rec}}$ is the recurrent weight matrix, $\boldsymbol{W}_{\mathbf{in}}$ is the input weight matrix, and $\boldsymbol{u}$ is the input. The mapping function $\boldsymbol{f}$ was set to the hyperbolic tangent. $\boldsymbol{W}_{\mathbf{rec}}$ was defined as a random matrix with connection density d, and $\boldsymbol{W}_{\mathbf{in}}$ was defined as a random matrix scaled by a parameter a_{in} (each element of $\boldsymbol{W}_{\mathbf{in}}$ ranges from $-a_{\mathrm{in}}$ to $+a_{\mathrm{in}}$). We fixed the reservoir size $N = 40$ and used a grid search to determine the optimal parameters, including the spectral radius of $\boldsymbol{W}_{\mathbf{rec}}$, the connection density d, and the scaling factor a_{in} of $\boldsymbol{W}_{\mathbf{in}}$. To account for performance variation due to the randomness of the weight matrices, each condition was tested 500 times, and the average root mean square error (RMSE) for the 10 step-ahead prediction task was used as the performance score. From the grid search results, as shown in Figure S16, the optimal parameters were determined to be d=0.42, r=0.48, and a_{in}=0.3.

In the computation load versus RMSE plot shown in Figure 7g, these parameters were fixed while varying N. For each N, the task was executed 500 times, and the average RMSE was plotted. The computational load in the ESN is quantified as the number of floating-point operations (FLOPs) required for inference, as shown in Equations S2 and S3. These represent the operations required for reservoir state evolution (equation S28) and the linear combination in the readout network (equation 1), respectively:

$$\text{Reservoir layer：FLOPs} = 2N^2 + 2N_uN + N - N_u \tag{S29}$$

$$\text{Readout layer：FLOPs} = 2N_yN - N_y + 1 \tag{S30}$$

Here, N_y and N_u denote the output and input dimensions, respectively. Equations S29 and S30 describe the FLOPs per discrete time step, and their sum represents the total FLOPs required for inference in the ESN.

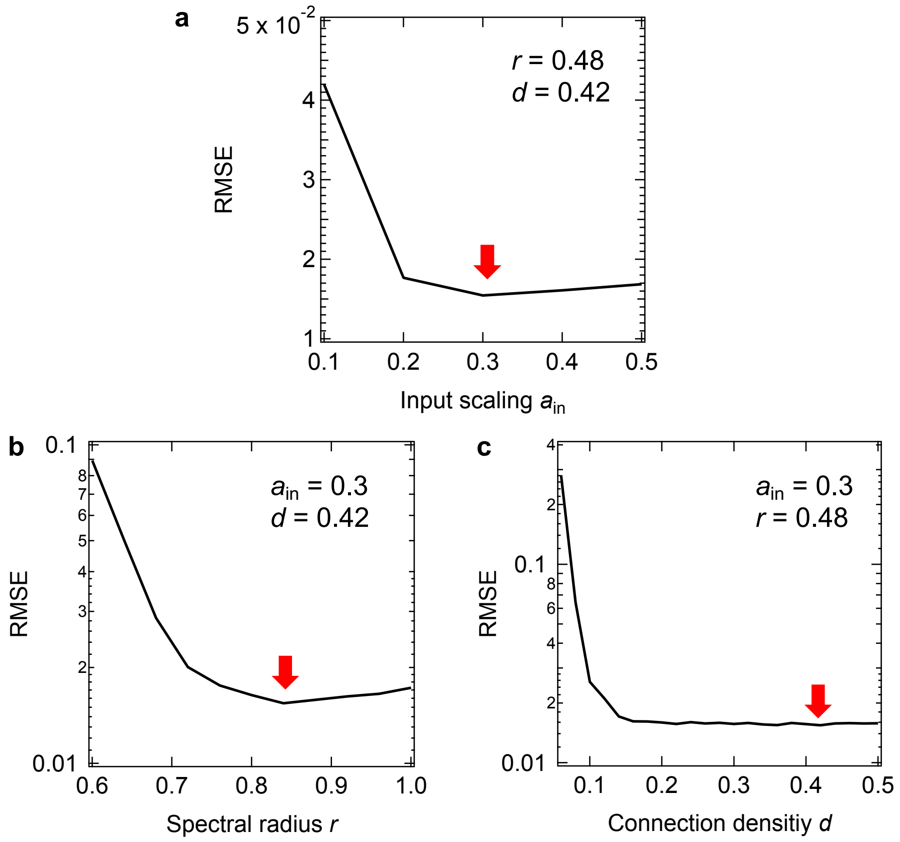

Figure S16: Grid search for ESN optimization. Test error dependence on (a) a_{in}, (b) r, and (c) d for the 10-step-ahead prediction task of the MG equation. The red arrows indicate the optimal conditions.

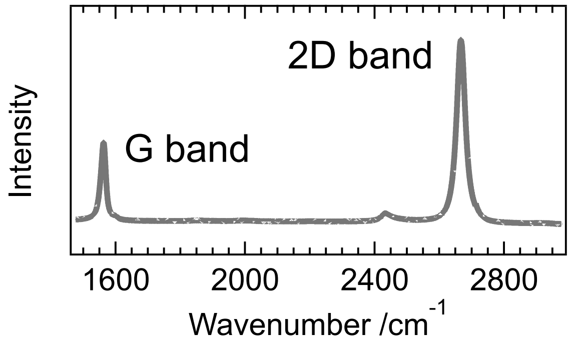

Figure S17: Raman spectrum of the graphene channel. The intensity ratio of the G band to the 2D band confirms that the graphene channel is monolayer[8)].

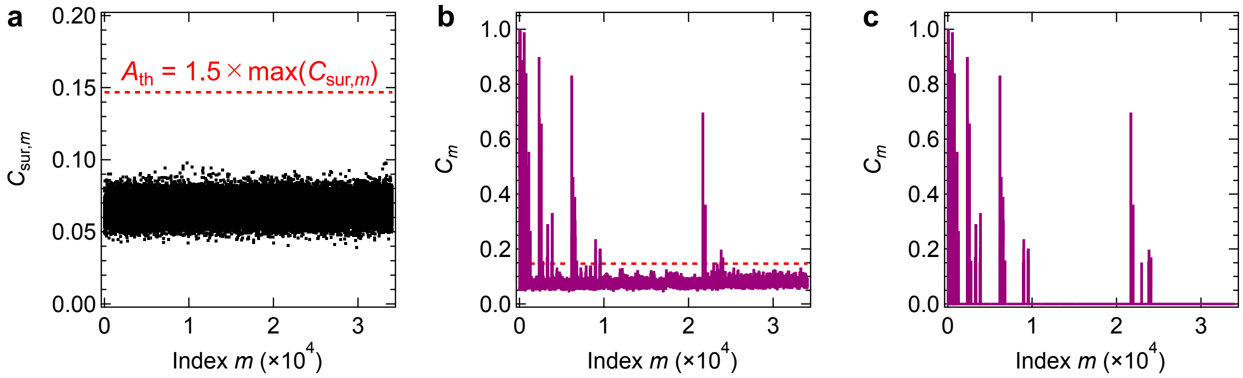

Figure S18: Surrogate method for IPC calculation. (a) Distribution of surrogate capacities $C_{\mathrm{sur},m}$ under conditions T=100 μs and d_{in}=2. The threshold A_{th} was set as 1.5 times the maximum surrogate capacity. (b) Component-wise capacities C_m for all indices under the same conditions. The red line indicates the threshold A_{th} obtained in (a). (c) C_m values for all indices after setting values below the threshold to zero. The IPC values presented in the main text were filtered using this procedure across all conditions to prevent overestimation[9)].

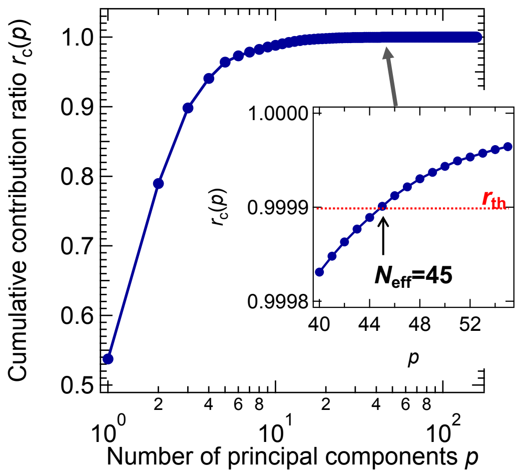

Figure S19: Calculation method of effective reservoir size N_{eff} using PCA. The plot shows the number of principal components p and the cumulative contribution ratio $r_c(p)$ under conditions T=50 µs and d_{in}=2. The inset expands the region near $r_c(p)$=100%, where the smallest p that exceeds the threshold r_{th} is defined as N_{eff}.

Supplementary references

1) Arihori, K., Ogawa, M., Souma, S., Sato-Iwanaga, J. and Suzuki, M.-A., "Transient performance analysis of graphene FET gated via ionic solid by numerical simulations based on tight-binding method and Nernst--Planck--Poisson equations," J. Appl. Phys. 130, 084302 (2021).
2) Gerischer, H., McIntyre, R., Scherson, D. & Storck, W. Density of the electronic states of graphite: derivation from differential capacitance measurements. *J. Phys. Chem.* **91**, 1930–1935 (1987).
3) Kornyshev, A. A. Double-layer in ionic liquids: paradigm change? *J. Phys. Chem. B* **111**, 5545–5557 (2007).
4) Lockett, V., Sedev, R., Ralston, J., Horne, M. & Rodopoulos, T. Differential capacitance of the electrical double layer in imidazolium-based ionic liquids: influence of potential, cation size, and temperature. *J. Phys. Chem. C.* **112**, 7486–7495 (2008).
5) Yamaguchi, T. et al. Low-temperature transport properties of holes introduced by ionic liquid gating in hydrogen-terminated diamond surfaces. *J. Phys. Soc. Jpn.* **82**, 074718 (2013).
6) Uesugi, E., Goto, H., Eguchi, R. et al. Electric double-layer capacitance between an ionic liquid and few-layer graphene. *Sci. Rep.* **3**, 1595 (2013).
7) Lukoševičius, M., & Jaeger, H. Reservoir computing approaches to recurrent neural network training. *Comput. Sci. Rev.* **3**, 127-149 (2009).
8) Ferrari, A. C., et al. Raman spectrum of graphene and graphene layers. *Phys. Rev. Lett.* **97**, 187401 (2006).
9) Tsunegi, S., et al. Information Processing Capacity of Spintronic Oscillator. *Adv. Intell. Syst.* **5** 2300175 (2023).
